# Dual disruption of aldehyde dehydrogenases 1 and 3 promotes functional changes in the glutathione redox system and enhances chemosensitivity in nonsmall cell lung cancer

**DOI:** 10.1038/s41388-020-1184-9

**Published:** 2020-02-03

**Authors:** Rocio Rebollido-Rios, Geoffroy Venton, Sara Sánchez-Redondo, Carmela Iglesias i Felip, Guy Fournet, Elena González, Wilber Romero Fernández, Dasiel Oscar Borroto Escuela, Barbara Di Stefano, Reinier Penarroche-Díaz, Guillaume Martin, Ismail Ceylan, Regis Costello, Mileidys Perez-Alea

**Affiliations:** 1 Faculty of Medicine and University Hospital of Cologne, Department I of Internal Medicine, University of Cologne, Cologne, Germany; 2 Service of Haematology and Cellular Therapy, Centre Hospitalier Universitaire La Conception, Marseille, France; 3 Microenvironment and Metastasis Laboratory, Molecular Oncology Programme. Spanish National Cancer Research Centre (CNIO), Madrid, Spain; 4 Anatomy Pathology Department, Vall d’Hebron Hospital, Barcelona-UAB, Barcelona, Spain; 5 Institut de Chimie et Biochimie Moléculaire et Supramoléculaire, UMR-CNRS 5246, Université de Lyon, Université Claude Bernard-Lyon1, Villeurbanne, France; 6 Oncology Research Program, Vall d’Hebron Research Institute, Barcelona, Spain; 7 Department of Cell and Molecular Biology, Computational Biology and Bioinformatics, Uppsala Universitet, Uppsala, Sweden; 8 Dichirons-Department of Surgical, Laboratory of Biology and Regenerative Medicine-Plastic Surgery- BIOPLAST, Oncological and Stomatological Sciences, University of Palermo, Palermo, Italy; 9 Reixmor, Barcelona, Spain; 10 Unit of Research in Cellular and Molecular Biology, Advanced Biodesign-ABD, Saint Priest, France

**Keywords:** Cancer metabolism, Cancer therapeutic resistance, Targeted therapies

## Abstract

Aldehyde dehydrogenases *(*ALDHs*)* are multifunctional enzymes that oxidize diverse endogenous and exogenous aldehydes. We conducted a meta-analysis based on The Cancer Genome Atlas and Gene Expression Omnibus data and detected genetic alterations in *ALDH1A1, ALDH1A3*, or *ALDH3A1*, 86% of which were gene amplification or mRNA upregulation, in 31% of nonsmall cell lung cancers (NSCLCs). The expression of these isoenzymes impacted chemoresistance and shortened survival times in patients. We hypothesized that these enzymes provide an oxidative advantage for the persistence of NSCLC. To test this hypothesis, we used genetic and pharmacological approaches with DIMATE, an irreversible inhibitor of ALDH1/3. DIMATE showed cytotoxicity in 73% of NSCLC cell lines tested and demonstrated antitumor activity in orthotopic xenografts via hydroxynonenal-protein adduct accumulation, GSTO1-mediated depletion of glutathione and increased H_2_O_2_. Consistent with this result, ALDH1/3 disruption synergized with ROS-inducing agents or glutathione synthesis inhibitors to trigger cell death. In lung cancer xenografts with high to moderate cisplatin resistance, combination treatment with DIMATE promoted strong synergistic responses with tumor regression. These results indicate that NSCLCs with increased expression of ALDH1A1, ALDH1A3, or ALDH3A1 may be targeted by strategies involving inhibitors of these isoenzymes as monotherapy or in combination with chemotherapy to overcome patient-specific drug resistance.

## Introduction

The aldehyde dehydrogenase (ALDH) family is a superfamily of intracellular enzymes that oxidize numerous diverse physiologically and pathophysiologically relevant aldehydes to their corresponding nontoxic carboxylic acids [1, 2]. Whereas ALDH family members play primarily cytoprotective biological roles via detoxification of aldehydes, they also modulate cell proliferation, differentiation and survival [3, 4].

In nonsmall cell lung cancer (NSCLC), which accounts for 85% of lung cancers, increasing evidence suggests that ALDHs play functional roles in tumorigenicity and drug resistance. Class 1 ALDH expression was found to correlate with invasive properties, chemoresistance and the in vivo ability to recapitulate original NSCLC [5, 6]. Activity of these enzymes was also reported to correlate with Notch expression, which drives a signaling pathway with alterations in ~30% of NSCLCs [7]. The STAT3-NF-ĸB/DDIT3/CEBPβ axis, one of the key oncogenic drivers in NSCLC, was found to regulate ALDH1A3 expression [8, 9]. More recent data indicated that stem cells located in airways may initiate cancer formation and cause the poor clinical outcome of NSCLC [10]. Notably, a main approach to isolate these tumor-initiating cells was based on their increased ALDH activity [11, 12].

The involvement of ALDHs in tumor initiation, therapeutic resistance, and malignant behavior has been extensively described in the literature [11–16]; however, the identity and therapeutic value of the specific isoform(s) contributing to these effects in particular tumor types—including lung cancer—remains largely elusive, partially due to the dearth of specific inhibitors and the misinterpretation of the extensively used ALDEFLUOR assay [17–22].

Here, we conducted a meta-analysis to identify ALDH isoforms with clinical and prognostic value in NSCLC. Using genetic and pharmacological targeting approaches, we showed that simultaneous inhibition of class 1 and class 3 ALDHs compromises glutathione (GSH) homeostasis. This condition ultimately results in severe oxidative damage and cell death. Finally, we demonstrated in vitro and in vivo that are independent of EGFR and KRAS status, ALDH1 and ALDH3 may be key therapeutic targets for NSCLC either alone or combined with ROS-inducing chemotherapeutic agents.

## Results

### Increased expression of ALDH1A1, ALDH1A3, or ALDH3A1 in NSCLC impacts chemotherapeutic responses and patient survival

**Fig. 1:**
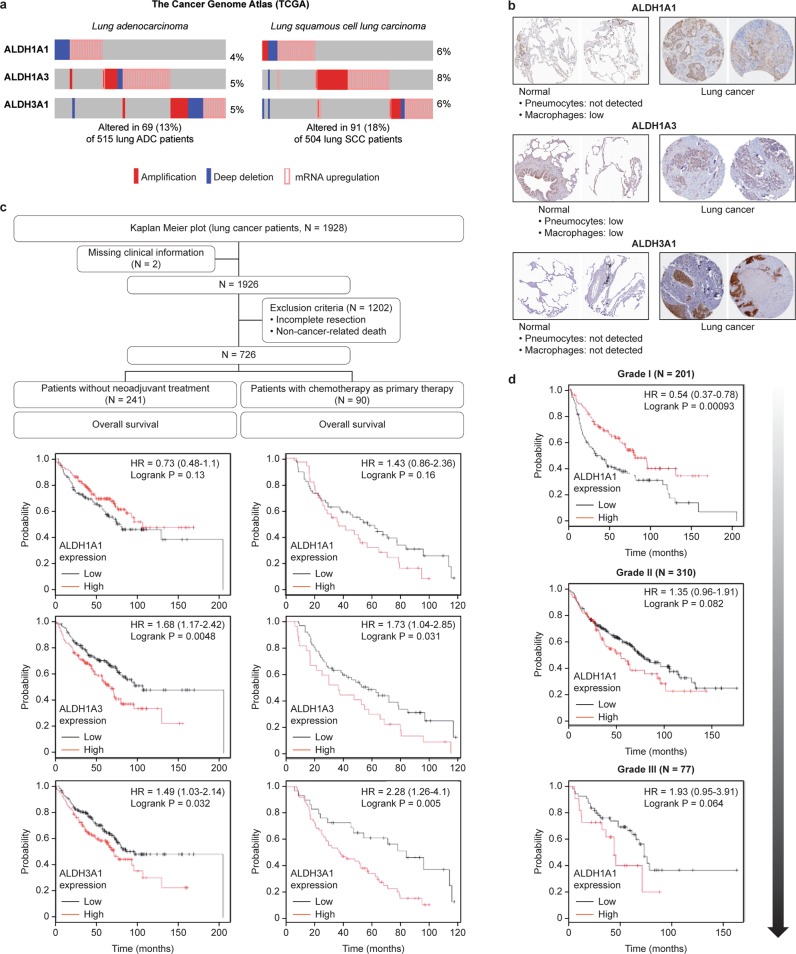
Expression of ALDH genes impacts the survival of NSCLC patients. **a** Frequencies of amplification (red bar), deletion (blue bar), and mRNA upregulation (empty bar) for *ALDH1A1*, *ALDH1A3,* and *ALDH3A1* in lung adenocarcinoma and lung squamous cell carcinoma, based on analysis of TCGA data (GISTIC2 analysis, cBioPortal). The percentages shown indicate the overall rates of gene amplification, upregulation and/or deletion in each subtype of NSCLC. The vertical aligned bars indicate samples from the same patient. **b** Representative protein expression profile for ALDHs based on immunohistochemistry using tissue microarrays. The figure shows normal pneumocytes exhibiting negative or low expression of ALDH1A1, ALDH1A3, and ALDH3A1 vs. medium to high protein expression in lung cancer. The images were obtained from the tissue section of the Human Protein Atlas project [24]. The annotated protein expression includes an evaluation of the staining intensity and percentage of stained cells. **c** Flow diagram summarizing the patient inclusion and exclusion criteria and Kaplan–Meier survival curves based on ALDH1A1, ALDH1A3, and ALDH3A1 expression. The vertical symbols represent censored cases. **d** Prognostic impact of ALDH1A1 expression on OS according to tumor grade.

First, we searched for alterations in the expression of genes of the ALDH family in the two major histopathological subtypes of NSCLC, lung adenocarcinoma (ADC) and squamous cell carcinoma (SCC), by analyzing RNA sequencing (RNAseq) data in datasets from The Cancer Genome Atlas (TCGA) (cBioPortal) [23]. We found that 13% of ADC (*N* = 515) and 18% of SCC (*N* = 504) patients carried transcriptional alterations in *ALDH1A1, ALDH1A3*, or *ALDH3A1* with a mutually exclusive tendency, suggesting that these genes confer similar functional effects (Fig. 1a). Overall, of the 158 NSCLC patients carrying alterations in any of these ALDH isoenzymes, 86% harbored either gene amplification or mRNA upregulation. The transcriptional alterations observed in these isoenzymes reflect the protein-level differences reported in Human Protein Atlas platform in normal vs. tumor tissue, changing from undetected or low staining in normal pneumocytes to moderate or intense staining in tumor tissues [24] (Fig. 1b).

Cytotoxic chemotherapy retains a major role in the management of advanced NSCLC [25]. Chemotherapy can be used before surgery to reduce the tumor size (neoadjuvant chemotherapy), after surgery in resected stage II and III NSCLCs or in stage III and IV lung cancers that cannot be removed surgically. Given the reported association of high ALDH activity with tumor-initiating cells and chemotherapeutic drug resistance [11, 13, 15], we next investigated the influence of *ALDH1A1*, *ALDH1A3*, and *ALDH3A1* mRNA expression on the survival of patients treated with or without chemotherapy, according to data in public NSCLC datasets from the TCGA and Gene Expression Omnibus (GEO) databases.

Patients with noncancer-related death, incomplete resection (R1), or missing clinical/pathological information were generally excluded from the analysis. We first analyzed the subset of patients with resected tumors who did not receive neoadjuvant chemotherapy; these patients were commonly early-stage patients. Overall survival (OS) analysis of 241 eligible patients revealed that patients with high *ALDH1A3* or *ALDH3A1* expression had significantly worse survival than those with low *ALDH1A3* or *ALDH3A1* expression (*P* = 0.005 and *P* = 0.032, log-rank test; Fig. 1c left, Table 1). We next evaluated OS in the subset of patients who received chemotherapy as primary therapy. Analysis of the 90 patients meeting these criteria showed poor prognosis in the group of patients with high *ALDH1A3* and *ALDH3A1* expression (*P* = 0.031 and *P* = 0.005, log-rank test; Fig. 1c right, Table 1), suggesting that ALDH1A3 and ALDH3A1 may affect not only early-stage patient prognosis but also the tumor response to chemotherapy.

**Table 1:** Median OS times of NSCLC patients according to the expression of *ALDH1A1*, *ALDH1A3* and *ALDH3A1.*

Median overall survival (months)					
Gene expression	NSCLC nontreated with neoadjuvant therapy (*N* = 241)	NSCLC treated with chemotherapy (*N* = 90)	Grade I (*N* = 201)	Grade II (*N* = 310)	Grade III (*N* = 77)
ALDH1A1^Low^	75	53.3	32.6	62	77.6
ALDH1A1^High^	95 (*P* = 0.130)	37 (*P* = 0.160)	79 (*P* = 0.001)	36 (*P* = 0.062)	32 (*P* = 0.064)
ALDH1A3^Low^	106	62	74	78.9	75.7
ALDH1A3^High^	68 (*P* = 0.005)	36 (*P* = 0.031)	48 (*P* = 0.139)	44.4 (*P* = 0.0522)	56.8 (*P* = 0.280)
ALDH3A1^Low^	96	77.6	68.6	96.1	65
ALDH3A1^High^	70.6 (*P* = 0.032)	32 (*P* = 0.005)	52 (*P* = 0.282)	65.2 (*P* = 0.062)	26.9 (*P* = 0.05)

For ALDH1A1, and only in the cohort treated with chemotherapy, patients with high ALDH1A1 expression showed shorter median OS times than patients with low ALDH1A1 expression (37 vs. 53 months, respectively; Fig. 1c right, Table 1). Though this trend was not statistically significant (*P* = 0.16), the tendency in the data was consistent with that in a previous study reporting a significant correlation between high ALDH1A1 expression and poor prognosis in patients with advanced NSCLC treated with induction chemotherapy [26].

ALDH1A1 has been associated with CSC populations in many tumor types [27]. Consistent with this observation, in patients with available tumor-grade characterization data and independent of treatment regimen (*N* = 588), we found survival differences based on ALDH1A1 expression and the histological grade of NSCLC (Fig. 1d, Table 1). While high ALDH1A1 expression was associated with prolonged survival of patients with well-differentiated tumors, an inverse trend toward worse OS with high ALDH1A1 expression was seen in patients with moderate- and poorly differentiated tumors.

To verify whether the expression of *ALDH1A1*, *ALDH1A3,* or *ALDH3A1* was related to other clinicopathological variables, a crosstab was subsequently generated (Table 2). We found no statistically significant associations between the expression of *ALDH1A1*, *ALDH1A3*, or *ALDH3A1* and age, sex, or tumor size. Interestingly, high expression of *ALDH1A1* was associated with nonsmoking status and lung squamous carcinoma. High *ALDH1A3* also showed a significant association with a history of no tobacco use and was associated with the ADC histological type, early-stage tumors and tumors without lymph node metastasis. *ALDH3A1* was highly expressed in lung SCC and in well- and moderately differentiated tumors.

**Table 2:** Associations between ALDH1A1, ALDH1A3, and ALDH3A1 expression and clinicopathological parameters.

Parameters (*N*)	*ALDH1A1*		*ALDH1A3*		*ALDH3A1*	
	High/Low	*P* value	High/Low	*P* value	High/Low	*P* value
Age		*0.06*		*0.06*		*0.75*
<65 (1042)	584/458		625/417		596/446	
≥65 (960)	497/463		536/424		556/404	
Sex		*0.33*		*0.79*		*0.1*
Male (1393)	729/664		708/685		814/579	
Female (816)	409/407		420/396		447/369	
Histology		2.80E−13*		2.20E−16*		2.69E−16*
Adenocarcinoma (1381)	625/756		859/522		711/670	
Squamous (917)	558/359		410/507		629/288	
Smoking history		1.20E−03*		1.44E−05*		*0.61*
Nonsmoker (245)	159/86		192/53		152/93	
Smoker (993)	529/464		635/358		596/397	
Grade		*0.29*		*0.8*		2.06E−03*
I–II (384)	207/177		274/110		248/136	
III-IV (321)	186/135		232/89		170/151	
Stage		*0.29*		3.53E−03*		*0.90*
I (757)	410/347		473/284		492/265	
II-III-IV (476)	243/233		257/219		307/169	
Lymph node affected		*0.19*		7.16E−03*		*0.08*
No (690)	341/349		432/258		387/303	
Yes (576)	306/270		280/296		294/282	
Tumor size		*0.57*		*0.7*		*0.53*
≤3 cm (368)	195/173		212/156		209/159	
>3 cm (790)	404/386		465/325		432/358	

*N* number of patients.

*P < 0.05.

### NSCLC cells are sensitive to DIMATE-mediated inhibition of class 1 and class 3 ALDH activity

**Fig. 2:**
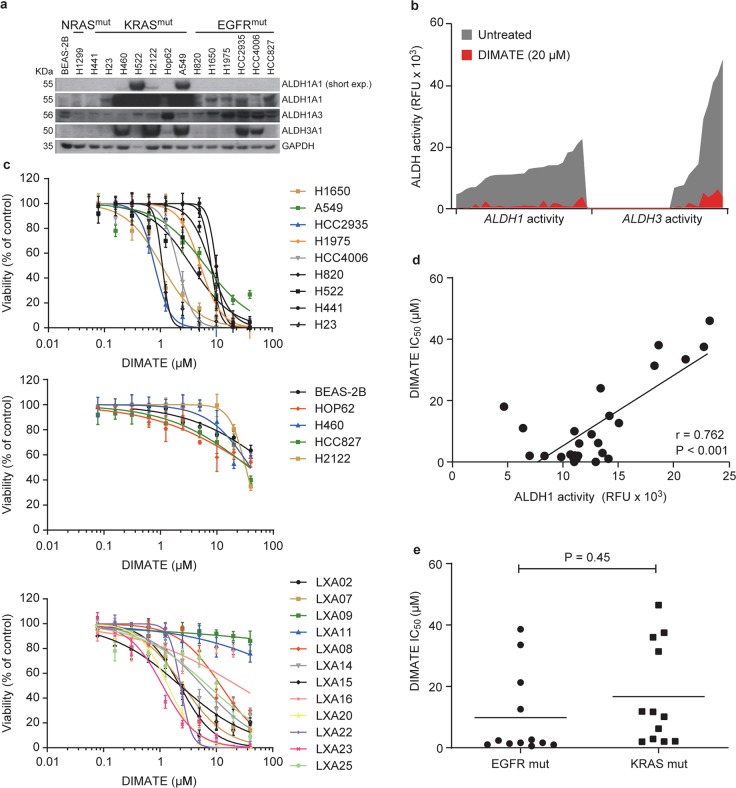
DIMATE affects the viability of NSCLC cells independent of their genetic background. **a** Immunoblots showing the amounts of ALDH1A1, ALDH1A3, and ALDH3A1 in normal human bronchial epithelial BEAS-2B cells and 14 NSCLC cell lines. GAPDH was used as the loading control. **b** Representative changes in ALDH1 and ALDH3 activity in an expanded panel of 26 NSCLC cell lines, including the cell lines in **a** and 12 xenograft-derived NSCLC primary cell lines (LXA), untreated or treated with the indicated dose of DIMATE. Data are plotted in increasing order according to the registered endogenous ALDH activity for each NSCLC cell line, i.e., from lower to higher mean values. A continuous connecting line was drawn to better illustrate the inhibition of the signal in the presence of DIMATE. ALDH activity was measured using a fluorometric enzymatic assay and two substrate probes (SEF0025 and SEF0013) with preferential affinity for ALDH class 1 and ALDH class 3 molecules, respectively (see the “Materials and methods” section for experimental details). **c** Dose-response curves for cell viability in the panel of 26 cell lines treated for 72 h with increasing doses of DIMATE. DIMATE-sensitive NSCLC cell lines are grouped in the upper plot; resistant cell lines, including the normal BEAS-2B line, are presented in the middle plot; and xenograft-derived NSCLC cells (LXA) are grouped in the lower plot. The error bars indicate the SDs (*N* = 4). **d** Graph showing the Pearson correlation between endogenous cellular ALDH1 activity and the IC_50_ values of DIMATE in the NSCLC cells in **c**. **e** Graphs showing the IC_50_ values of DIMATE in the NSCLC cell lines according to the mutational status. The horizontal bars indicate the mean values.

To evaluate the possible effects of inhibiting ALDH1A1, ALDH1A3, and ALDH3A1 isoenzymes, we used 14 NSCLC cell lines with different oncogenic driver alterations and the noncancerous cell line BEAS-2B for comparison. In all cell lines, the transcriptional profiles of *ALDH1A1*, *ALDH1A3*, and/or *ALDH3A1* showed mRNA upregulation across the different NSCLC lines and compared to BEAS-2B cells (Fig. S1a, b). These differences were reflected at the protein level and encompassed both the high expression and mutually exclusive pattern observed for the three ALDH isoenzymes in the patient cohort (Figs. 1a and 2a), and in NSCLC tumor tissues vs. normal cells (Figs. 1b and 2a).

We previously showed that 4-dimethylamino-4-methylpent-2-ynthioic acid S-methyl ester (DIMATE) is an enzyme-activated and irreversible inhibitor of class 1 and class 3 ALDHs [20, 28, 29]. Using biochemical assays, we demonstrated that DIMATE efficiently suppresses ALDH1 and ALDH3 enzymatic activities in an expanded panel of 26 NSCLC cell lines that included the 14 NSCLC cell line panel and 12 primary cell lines from patient-derived xenografts (Fig. 2b). In addition, DIMATE induced a significant dose-dependent reduction in viability in 19 of the 26 NSCLC cell lines tested (≥50%, *P* < 0.001; Fig. 2c, Table S1). BEAS-2B cells, which were used as normal counterparts, and showed relatively low ALDH1A3 protein levels and undetected ALDH1A1 and ALDH3A1 levels, were highly resistant to DIMATE-induced cell death (half maximal inhibitory concentration (IC_50_) = ~50 µM) (Fig. 2a, c). Overall, the effect of DIMATE on NSCLC cell viability and the endogenous ALDH activity in untreated cells were positively correlated, suggesting specific interaction of the drug with its target (Fig. 2d). We found no relationships between the cellular response to DIMATE and EGFR- or KRAS-mutated genotypes (Fig. 2e).

### HNE-protein adduct accumulation and redox imbalance are the primary causes of DIMATE-induced cell death in vitro

**Fig. 3:**
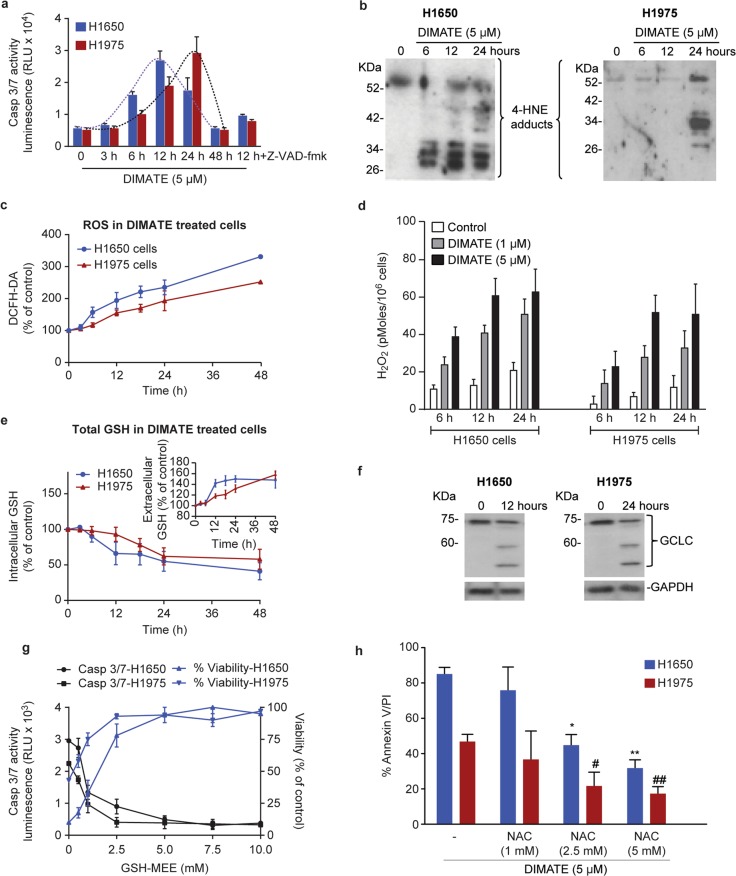
DIMATE elicits GSH depletion and oxidative stress-mediated apoptosis. **a** Bioluminescence measurements of caspase 3/7 activity in cells treated with 5 µM DIMATE at the indicated times. The pancaspase inhibitor Z-VAD-fmk was used as the negative control to confirm the specificity of the DIMATE-induced signal. Data are expressed as relative light units (RLU). Error bars ± SEM. (*N* = 3). **b** Immunoblots showing the accumulation of HNE adducts over time in H1650 and H1975 cells treated as described in **a**. **c** Total intracellular ROS levels in H1650 and H1975 cells exposed to 5 µM DIMATE for the indicated times. Error bars ± SD (*N* = 3). **d** Quantification of H_2_O_2_ in H1650 and H1975 cells exposed to DIMATE for the indicated times. The values are the averages of two independent experiments. **e** Time course quantification of intracellular and extracellular GSH in H1650 and H1975 cells treated with 5 μM DIMATE for the indicated times. The results are expressed relative to untreated controls and are shown as the means ± SDs. (*N* = 2). **f** Expression of full length and cleaved GCLC, as assessed by immunoblotting. GAPDH was used as the loading control. **g** Caspase 3/7 activity in H1650 and H1975 cells treated with 5 μM DIMATE for 12 h in the presence of increasing concentrations of GSH-MEE (left axis). The right axis indicates the percentage of cell viability determined simultaneously for the same cells. The mean ± SD is shown (*N* = 3). **h** Graphs indicating the percentage of apoptotic H1650 and H1975 cells treated with DIMATE as described in **g** in the presence of various concentrations of the antioxidant NAC. Cell death was assessed by flow cytometry. The error bars indicate the SDs (*N* = 3); *P < 0.01, **P < 0.001.

The lung ADC cell lines H1650 and H1975 were selected for further analysis given their resistance to EGFR-targeted therapies and their high sensitivity to DIMATE (IC_50_ = 1.36 µM and IC_50_ = 1.62 µM, respectively). In these cells, DIMATE induced a time-dependent increase in caspase 3/7 activity and apoptotic cell death. (Fig. 3a and S2a). DIMATE also readily caused accumulation of hydroxynonenal (HNE) and malondialdehyde (MDA), two apoptogenic aldehydes that are detoxified by ALDH1 and ALDH3, over time (Fig. 3b and S2b) [3]. This finding suggested a relationship between the formation of new HNE and MDA adducts and the onset of apoptosis. As HNE and MDA accumulated, cells exhibited increased levels of reactive oxygen species (ROS), particularly H_2_O_2_ (Fig. 3c, d), and a rapid drop in intracellular levels of reduced GSH, a crucial molecule for the detoxification of H_2_O_2_ (Fig. 3e) [30]. GSH also participates in the elimination of HNE through the formation of GSH-HNE conjugates that are exported from cells [31]. Accordingly, after 48 h of exposure to the inhibitor and following the accumulation of 4-HNE, total intracellular GSH levels were decreased by almost 50%, while GSH increased in the extracellular media (Fig. 3e). Moreover, HNE coimmunoprecipitation combined with mass spectrometry analysis revealed that GSH S-transferase omega-1 (GSTO1) associated with HNE in DIMATE-treated cells (Table S2). GSTO1 catalyzes nucleophilic conjugation of GSH with a wide spectrum of electrophiles, including HNE, when the cellular levels of HNE are abnormally elevated [32]. Thus, depletion of GSH could result from active cellular efflux of reduced GSH-HNE adducts.

In addition to GSTO1, other proteins were identified to associate with HNE in DIMATE-treated cells (Table S2). These proteins included l-lactate dehydrogenase, pyruvate kinase isoenzymes M1/M2, malate dehydrogenase, succinyl-CoA ligase, electron transfer flavoprotein and septin-7, which are involved in several cellular events, including energy metabolism, redox homeostasis and cell cycle progression.

Western blot analysis of the catalytic subunit of glutamate cysteine ligase (GCLC), the rate-limiting enzyme in GSH biosynthesis, revealed cleavage of GCLC in DIMATE-treated cells and correlated temporally with the induction of caspase 3/7 activity (Fig. 3f, a). The observed size of the cleaved GCLC fragment was 60 kD, the same as that reported for the longer fragment resulting from caspase-3-mediated processing of GCLC [33].

Next, we investigated whether maintaining intracellular levels of GSH could impact DIMATE sensitivity. DIMATE-induced apoptosis was dose-dependently inhibited by GSH-monoethyl ester (GSH-MEE), a membrane-permeable GSH analog (Fig. 3g). Similarly, N-acetyl cysteine (NAC), a synthetic precursor of intracellular cysteine and GSH, dose-dependently improved cell viability (Fig. 3h), confirming the importance of GSH depletion during the cellular response to DIMATE.

### DIMATE suppresses tumor growth in an orthotopic human lung cancer xenograft model

**Fig. 4:**
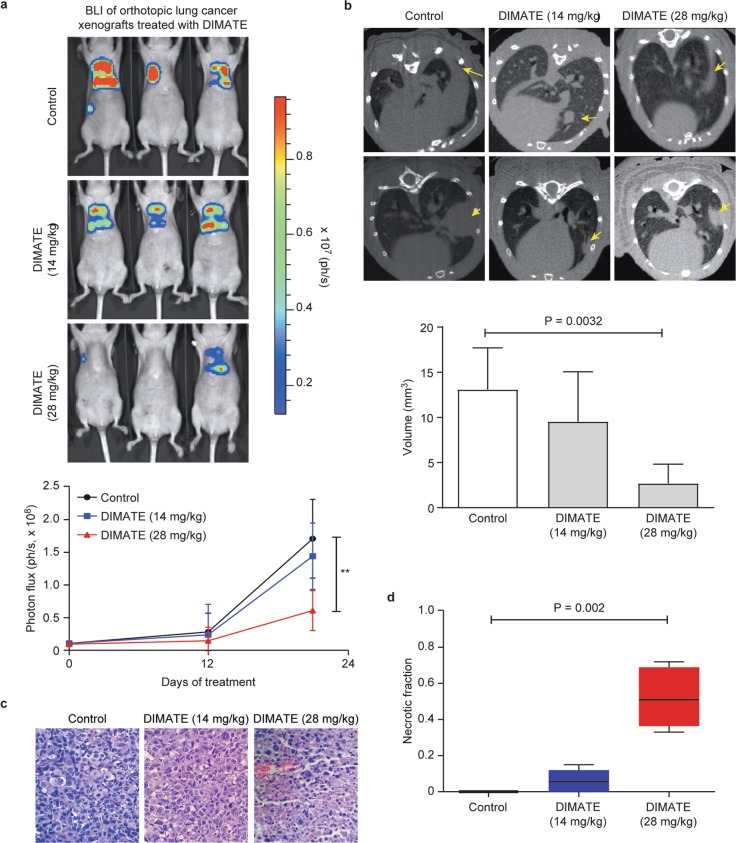
Tumor growth-inhibitory effect of DIMATE in orthotopic H1975 xenografts. **a** Representative bioluminescence imaging of tumor growth in H1975/luc xenografts treated with DIMATE or vehicle control on day 21 of treatment. The lower graph shows the quantification of tumor growth as the average photon flux emitted from each group at different time points. **P < 0.001 (28 mg/kg DIMATE group vs. control group). **b** Representative micro-CT images showing the lung anatomy of mice with orthotopic H1975 xenografts treated at two different concentrations of DIMATE as described in **a**. Macroscopic tumor lesions in the lungs are indicated with arrows. The lower graph represents the tumor volume calculated from CT scans. Error bars ± SD (*N* = 6). **c** Representative images of lungs harvested at necropsy and stained with hematoxylin and eosin (H&E) (×20 magnification). **d** Quantification of tumor necrosis by densitometry. The boxes extend from the 25th to the 75th percentile; the lines indicate median values, and the whiskers indicate the range of values.

To evaluate the anticancer activity of DIMATE in vivo, we used an orthotopic model of lung cancer. H1975/Luc cells were surgically inoculated into the lung parenchyma of athymic mice. The implanted cells formed primary nodules in the lung, which expanded with time into the contralateral lung and mediastinal tissues. After 3 weeks of treatment with 14 mg/kg and 28 mg/kg DIMATE, the bioluminescence signal was reduced by 18% and 64%, respectively, compared to that in control mice (*P* = 0.33; *P* < 0.01) (Fig. 4a). Consistent with this finding, the computed tomography (CT) images showed 27% and 79% tumor growth inhibition in animals administered 14 mg/kg and 28 mg/kg DIMATE, respectively, compared to control animals (*P* = 0.30 and *P* < 0.005; Fig. 4b).

Histopathological analysis of lung tissues corroborated neoplastic proliferation, with large, solid glandular growth, and numerous pleomorphic cells (Fig. 4c). Notably, tumor necrosis was dose-dependently enhanced in DIMATE-treated animals (Fig. 4d). According to clinicopathological parameters and associated pathological events in other organs and tissues, we found no apparent toxic effects of DIMATE in treated animals.

### Alteration of the tumor redox balance enhances the sensitivity of NSCLC cells to DIMATE

**Fig. 5:**
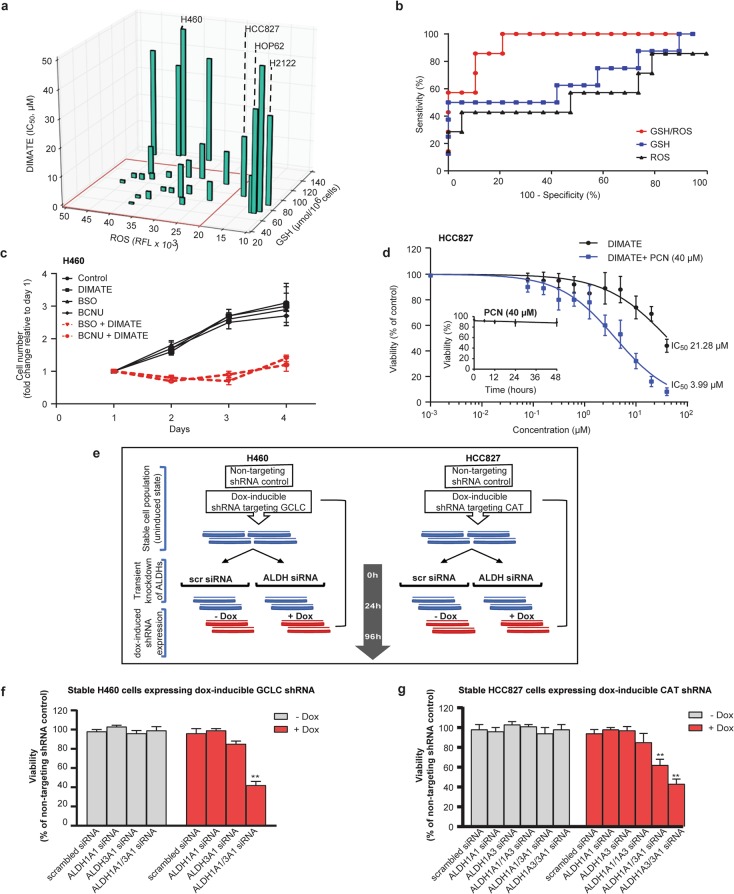
The response of NSCLC to ALDH1 and ALDH3 inhibition is synergistically enhanced by ROS-inducing agents or inhibitors of the GSH cellular antioxidant system. **a** Three-dimensional plot showing the distribution of IC_50_ values for DIMATE and the total intracellular GSH and ROS levels in each of the 26 NSCLC cell lines. The square highlights cells showing high sensitivity to DIMATE, which are also the cells with high ROS levels and low to moderate GSH levels. **b** ROC curves for the response of NSCLC cells to DIMATE vs. ROS levels, total intracellular GSH levels and the ROS:GSH ratio. (AUC = 0.67, 0.56, 0.93, respectively). ROC receiver operating characteristic; AUC area under the ROC curve. **c** Relative viability of H460 cells treated with 15 µM DIMATE, 100 µM BSO or 50 µM BCNU alone and in the indicated combinations as a function of time. The drug vehicle was used as the control. A significant decrease in viability was observed in H460 cells treated with DIMATE plus BSO or DIMATE plus BCNU compared to cells treated with the control or compound alone (*P* < 0.001; *t*-test). The data are expressed as the mean fold changes in cell number relative to day 1 ± SDs (*N* = 3). **d** Dose-response curve for the viability of HCC827 cells exposed for 48 hours to DIMATE in the presence or absence of PCN. The IC_50_ values for the different conditions are provided in the graph. The inner panel shows that 40 μM PCN exhibited no cytotoxicity at the indicated incubation times. The error bars indicate the SD (*N* = 4). **e** Schematic representation of the strategy used to evaluate the viability of H460 and HCC827 cells when transient depletion of ALDH1 and/or ALDH3 was combined with the reduction in redox capacity induced by GCLC shRNA or CAT shRNA, respectively. **f**, **g** Viability of stable H460 cells expressing dox-inducible GCLC shRNA and HCC827 cells expressing dox-inducible CAT shRNA as described in **e** with ALDH1 or ALDH3 depletion as indicated in the *x* axis at the 96-h time point. The data are expressed as percentages relative to control cells. The error bars indicate the SDs. (*N* = 3); **P < 0.005.

In our panel of 26 NSCLC cell lines expressing ALDH class 1 and/or 3 molecules, cells with high ROS levels and concomitant low to moderate GSH levels were significantly more susceptible to DIMATE-induced cell death (average IC_50_ = 4.05 ± 3.83; *P* < 0.001) than cells with either low ROS levels or the highest GSH levels in the cohort (average IC_50_ = 30.40 ± 11.48) (Fig. 5a). Consistent with this finding, the endogenous ROS:GSH ratio had a good predictive ability to discriminate DIMATE-sensitive from DIMATE-resistant cells (AUC = 0.93; Fig. 5b).

To verify that DIMATE responses are influenced by the cellular redox state, we investigated the effect of two agents that inhibit the synthesis of GSH: l-buthionine-sulfoximine (BSO), an irreversible inhibitor of gamma-glutamylcysteine synthetase [34] and bis-chloroethylnitrosourea (BCNU), a GSH reductase inhibitor [35, 36], in H460 cells that are resistant to DIMATE (IC_50_ = 46.5 µM) and possess high GSH levels (136 µmol/10^6^ cells). While 15 µM DIMATE, 100 µM BSO, or 50 µM BCNU alone did not affect cell viability, the growth of cells treated with a combination of DIMATE and either BSO or BCNU decreased by approximately twofold (*P* < 0.001; Fig. 5c), accompanied by a significant increase in the apoptosis rate from 11% in cells treated with single agents to 36 and 44% in cells receiving DIMATE in combination with BSO or BCNU, respectively (*P* < 0.001; Fig. S3a).

Using a different approach to induce cellular redox imbalance, the cell lines HCC827, Hop62, and H2122, which showed poor responses to DIMATE and low endogenous levels of ROS, were exposed to pyocyanin (PCN). PCN induces oxidative stress partially due to its ability to increase intracellular H_2_O_2_ and superoxide levels [37]. In cells treated with noncytotoxic concentrations of PCN, DIMATE sensitivity was increased by approximately four- to fivefold (Fig. 5d and S3b).

Next, we validated that the cellular redox state determines the response of NSCLC cells to inhibition of ALDH 1 and 3 by genetically reducing the redox capacity in two NSCLC cell lines, H460 and HCC827. Cells were infected with tetracycline (Tet)-inducible system expressing either nontargeting short hairpin RNA (shRNA) control, shRNA targeting GCLC (in H460 cells) or shRNA targeting catalase (CAT) (in HCC827 cells) (Fig. S4a, b). GCLC knockdown decreased the intracellular levels of endogenous GSH by approximately half in H460 cells (Fig. S4c) without affecting cell viability (Fig. S4d), thus establishing a system with low GSH redox capacity. In HCC827 cells, catalase downregulation did not result in a direct increase in intracellular H_2_O_2_, as expected (data not shown) [38]; however, under treatment with exogenous H_2_O_2_ (100 µM), viability was significantly compromised only in catalase-depleted HCC827 cells (Fig. S4e), confirming that the capacity of these cells to clear H_2_O_2_ through catalase-mediated degradation was decreased. Catalase downregulation did not impact the viability of HCC827 cells (Fig. S4f).

In both H460 and HCC827 cells, transient knockdown of their corresponding highest-abundant ALDH class 1 molecule (ALDH1A1 in H460 and ALDH1A1 and ALDH1A3 in HCC827 cells) resulted in a compensatory increase in ALDH3A1 protein expression (Fig. S5a, b) but did not significantly affect cell viability (Fig. S5c, d). Only simultaneous knockdown of ALDH1/ALDH3A1 followed by doxycycline induction of shRNA targeting GCLC in H460 cells or catalase in HCC827 cells significantly reduced cell viability by 81.5% and 70.5%, respectively (Fig. 5e–g). Collectively, these data suggest that NSCLC cells with high expression of class 1 and/or class 3 ALDHs and poor responses to simultaneous inhibition of these isoenzymes could be sensitized by combining this inhibition with other compounds that affect cellular redox status.

### Synergistic effect of DIMATE and CDDP in the orthotopic mouse model of NSCLC

CDDP-based chemotherapy is indicated as a first-line treatment for NSCLC, though, responses to this drug are generally moderate [25]. The CDDP action mechanism includes the generation of nuclear DNA adducts together with its ability to increase total ROS by a mechanism that appears independent of nuclear DNA damage signaling [39, 40]. To further confirm the hypothesis that DIMATE treatment benefits from treatment with drugs affecting redox homeostasis, we evaluated the effectiveness of combining DIMATE with CDDP in vitro and in vivo.

**Fig. 6:**
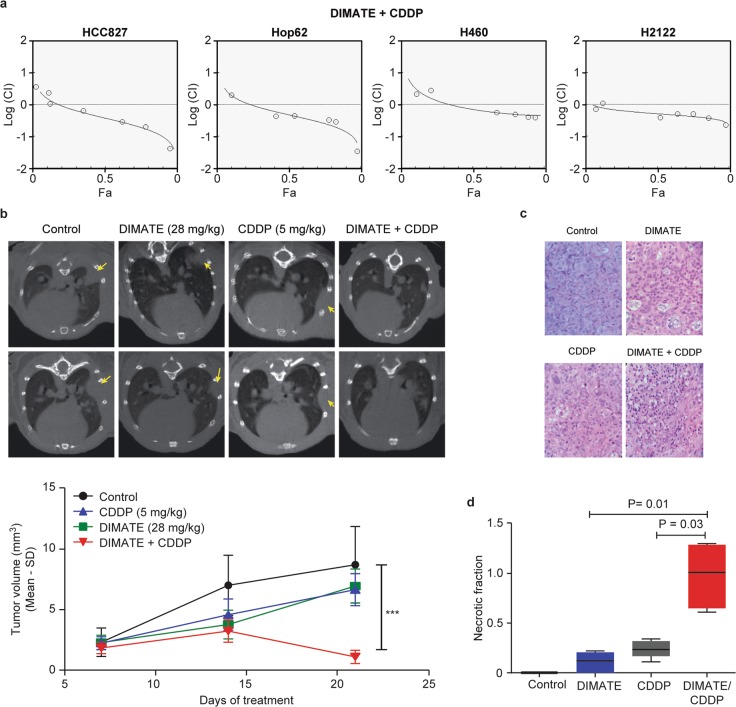
Synergistic inhibitory effect of DIMATE and CDDP in NSCLC cells. **a** CI curve analysis (Chou–Talalay plot) for DIMATE plus CDDP in HCC827, H460, Hop62, and H2122 cells, indicating synergy at medium and high doses. CI values less than, equal to, or greater than 1 indicate synergy, additivity, or antagonism, respectively. The effect level (Fa) indicates the fractional inhibition for each CI. **b** Representative micro-CT images showing the lung anatomy of mice with orthotopic HCC827 xenografts treated with either DIMATE, CDDP or DIMATE plus CDDP. Macroscopic tumor lesions in the lungs are indicated with arrows. The lower graph shows the tumor volumes calculated from CT scans at the indicated time points. **c** Representative images of pulmonary tumors at necropsy stained with H&E (20X magnification). **d** Quantification of tumor necrosis within tumor sections by densitometry.

In vitro, the extent of synergism (combination index (CI) values) obtained for DIMATE and CDDP at 50–95% drug effect (Fa) levels differed among the four resistant cell lines tested (HCC827, Hop62, H2122, and H460), but the combination showed stronger synergy at higher doses (Fa > 0.30, CI < 1) in all cell lines (Fig. 6a and Table S3).

Via a different approach and in HCC827 cells, which are resistant to both DIMATE and CDDP (IC_50_ = 24 and 15 µM, respectively), responses to DIMATE were compared among cells with low endogenous levels of ROS and cells with moderate and high levels of ROS induced by CDDP treatment. (Fig. S6a). The extent of apoptosis induced by DIMATE was proportional to the increase in the ROS level in the three different cell populations; the apoptosis rates were ~44%, 79% and 98% in cells with low, moderate and high ROS levels, respectively (Fig. S6a). No differences in ALDH1A1, ALDH1A3 and ALDH3A1 protein expression were observed between untreated and CDDP-treated cell subpopulations (Fig. S6b).

To evaluate the benefit in a preclinical setting, the efficacy of DIMATE plus CDDP was investigated in vivo in an HCC827 orthotopic lung cancer model. Mice receiving combination therapy showed rapid tumor regression and significantly higher tumor growth inhibition (*P* < 0.001) than control mice and mice receiving DIMATE or CDDP as monotherapy (Fig. 6b). Consistent with this result, histopathological examination of pulmonary tumor tissues showed the highest necrotic density in animals treated with DIMATE and CDDP in combination (0.98 for the combination vs. 0.11 and 0.24 for DIMATE and CDDP alone, respectively; Fig. 6c, d). Tumor necrosis was not evident in the controls.

## Discussion

The involvement of ALDH in tumor initiation, therapeutic resistance and malignant behavior has become a popular topic in cancer research (reviewed in [11–16]). Previous studies reported a significant correlation of class 1 ALDHs with poor clinical outcome in patients with NSCLC [5, 26, 41]. Although these initial studies could not separate the contribution of individual isoenzymes, the results underscored the clinical potential of ALDHs in lung cancer. More recently, ALDH1A1 and ALDH3A1 were reported to be highly expressed in NSCLCs, and both isoenzymes were found to be overexpressed in putative lung epithelial stem cell niches in tumors compared with normal lung tissues [6, 7]. These observations have generated interesting speculation on the relationship of ALDHs to lung cancer [10, 12].

Here, we described *ALDH1A1*, *ALDH1A3,* and *ALDH3A1* gains and mRNA upregulation to be associated with prognosis in lung ADC and SCC histological subgroups and provided supporting evidence that ALDH1A3 and ALDH3A1 could be a prognostic marker in NSCLC patients. In addition, we found that ALDH1A3 expression was associated with ADC histology, while ALDH3A1 was strongly associated with squamous histology. Consistent with our results, Shao et al. [9] found that ALDH1A3 was highly expressed in early-stage tumors and patients with a nonsmoking history. We also revealed a significant association of high ALDH1A1 with never-smoker status, identifying ALDH1A1 and ALDH1A3 as possibly related to new risk factors in the ~10–20% of lung cancers occurring in never smokers [42]. Interestingly, normal pneumocytes of smokers are reported to exhibit increased expression of ALDH1A1 compared with those of nonsmokers [6]. These data, with our findings, support the key role that ALDH1A1 plays in the response to xenobiotics (likely, tobacco, chemotherapy and other chemicals) and invite a careful interpretation of studies comparing ALDH1A1 expression in lung tumor vs. normal lung tissues considering smoking patterns and exposure to other toxic agents. Consistent with this observation and in partial contrast with three earlier studies reporting ALDH1A1 as a predictor of poor prognosis in NSCLC [7, 43, 44], our results using more restrictive exclusion criteria indicated that high levels of ALDH1A1 determine significantly better OS in patients with well-differentiated tumors and provide an overall minimal benefit in patients with early-stage tumors and no neoadjuvant treatment. Although this protective effect of ALDH1A1 was reversed in moderately and poorly differentiated tumors and in chemotherapy-treated patients, this trend was nonsignificant. ALDH1A1 is likely to be linked to a generally protective role in xenobiotic clearance in the lungs. These observations place the ALDH1A1 in a different category of ALDHs.

DIMATE is an irreversible inhibitor of class 3 and class 1 ALDHs (except ALDH 2) [28, 29]. Because of this dual effect, we selected this compound to investigate the pharmaceutical effect of simultaneous inhibition of ALDH 1 and 3 in NSCLC. Our study showed that DIMATE causes the accumulation of aldehydes and aldehyde-protein adducts that might compromise protein function and fate in cancer cells [31]. In parallel, intracellular levels of GSH decreased as GSH was engaged in active-enzyme mediated detoxification of the accumulated aldehydes. These early events triggered an amplification cycle of oxidative stress carried by (i) enhanced formation of ROS, particularly H_2_O_2_, which might be the source of new aldehydes due to lipid peroxide attack, [32] and (ii) GCLC cleavage, likely mediated by caspases, which would affect de novo synthesis of GSH, further enhancing the intracellular redox imbalance that precipitates cell death [45]. We demonstrated the in vivo efficacy of DIMATE in lung cancer orthotopic models. Notably, DIMATE can increase sensitivity to ROS-inducing agents, a criterion met by existing mainstay cancer therapies [46]. Specifically, we showed that CDDP and DIMATE were synergistic at reduced doses, suggesting a potential means for improving tumor responses to platinum-based antineoplastic agents and avoiding the toxicities of this drug class.

In conclusion, we show for the first time the benefit of using dual inhibitors of ALDH1 and ALDH3 as redox-disrupting agents in lung carcinomas and provide preclinical support for their use as monotherapies or in combination with other ROS-targeting drugs in NSCLCs, including chemoresistant tumors.

## Material and methods

### Cell lines and cell culture

A panel of 26 human NSCLC cell lines and the cell line BEAS-2B were obtained either from the American Type Culture Collection or from the laboratory of Dr Yokota Jun (Research Institute Germans-Trias Pujol, Spain), as detailed in Supplementary Table S1. Twelve patient-derived lung tumor xenograft cell lines (named LXA for Lung Xenograft ADC) were obtained from Oncotest (Freiburg, Germany), and are described in detail in Table S1. BEAS-2B cells were cultivated in BEGMBronchial Epithelial Cell Growth Medium (Lonza, Barcelona, Spain). The other cell lines were propagated in RPMI-1640 medium supplemented with 10% FBS, 100 U/mL penicillin and 100 μg/mL streptomycin. All cells were maintained in a humidified atmosphere containing 5% CO_2_ at 37 °C.

### Cell viability assays

Cells were seeded in 96-well plates and treated with serial dilutions of DIMATE (0.01–100 µM) with or without PCN (40 µM), BSO (100 µM), or BCNU (50 µM) for 12, 48, and 72 h and analyzed using alamarBlue dye (Thermo Fisher, Saint Herblain, France). Drug responses were quantified by the IC_50_ for each cell line and determined by nonlinear regression analysis of log dose-response curves. The cutoff value for DIMATE resistance was determined statistically (greater than the geometric mean IC_50_ + SD). To reverse DIMATE-induced cell death, cells were exposed to 10 µM DIMATE in the presence of GSH-MEE at different concentrations (1–10 mM). Cell viability was measured using the alamarBlue assay following the manufacturer’s instructions (Thermo Fisher).

### Immunoblotting

For protein expression analysis, cells were lysed in RIPA buffer containing phosphatase and protease inhibitors (Calbiochem, San Diego, CA, USA). Total protein extracts were separated via SDS-PAGE and transfer to polyvinylidene difluoride membranes (Bio-Rad electrophoresis and Trans-Blot turbo transfer systems, Marnes-la-Coquette, France). Immunodetection was performed using standard protocols [47]. Antibodies used in these studies are listed in Table S4.

### Analyses using NSCLC cells

To analyze ALDH activity, cells incubated with fluorescent probes derived from propional (10 µM, SEF0025) and benzoate (5 µM, SEF0013), two preferred substrates of ALDH1 and ALDH3, respectively [20]. Enzymatic reactions were performed in live cells at 37 °C for 30 min and read immediately using an Appliskan fluorescence microplate reader (ex = 530 nm; em = 600 nm).

H_2_O_2_ was measured using an Amplex UltraRedfluorometric assay (Invitrogen, Cailloux-sur-Fontaines, France), and Caspases-3 and -7 activity was estimated using a Caspase-Glo 3/7 assay (Promega, Charbonnieres-les-Bains, France). The pancaspase inhibitor Z-VAD-fmk (50 µM) was used as the experimental control.

Reduced and oxidized GSH levels were measured using a GSH/GSSG-Glo assay kit (Promega) following the manufacturer’s instructions. Acivicin (0.5 mM) was used to block degradation of extracellular GSH. Protein titration was performed with cell lysate by the BCA method after both extracellular and intracellular GSH measurements. Luminescence was read in a Synergy Mx luminometer (BioTek, Winooski, VT, USA) with a 1-min lag time and a 0.5 s/well read time.

### Small interfering RNA (siRNA) and shRNA assays

For transient experiments, cells were transfected with either a scrambled siRNA or different siRNAs targeting *ALDH1A1*, *ALDH1A3*, or *ALDH3A1* obtained from Invitrogen-Thermo Scientific (see Fig. S5e for sequences). For inducible transcriptional downregulation, cells were infected with the hu-GCLC or hu-CAT Tet-inducible lentiviral shRNA system (Dharmacon SMARTvector, Horizon, Cambridge, UK). The control vector used was a nontargeting DNA (Horizon). shRNA sequences are detailed in Table S5. For shRNA expression, the culture medium was supplemented daily with 5 mM doxycycline. Knockdown efficiency was evaluated by Wes, quantification of intracellular GSH levels and the cellular oxidative response to H_2_O_2_.

### CI analysis

DIMATE-CDDP interactions were analyzed using CI curves and CompuSyn software made available by Chou [48]. Cells were seeded in 96-well plates and treated with different concentrations of DIMATE/CDDP in combination using constant ratios for each drug. The drug effect level (Fa) was monitored at 48 h using the alamarBlue assay as described above.

### In vivo orthotopic xenograft mouse model

The institutional Animal Care and Use Committee at Vall d’Hebron Research Institute (VHIR, Barcelona, Spain) approved all animal protocols described in this study. Firefly luciferase-labeled H1975 (H1975/Luc) or HCC827 cells (1 × 10^6^) were surgically implanted into the lung parenchyma of female, athymic nude mice as previously described [49]. Animals were complete randomized into the following groups: control (*N* = 6), 14 mg/kg DIMATE (*N* = 6), and 28 mg/kg DIMATE (*N* = 6) for H1975/Luc xenografts; and control (*N* = 6), 28 mg/kg DIMATE (*N* = 6), 5 mg/kg CDDP (*N* = 6) and DIMATE/CDDP (*N* = 6) for HCC827 xenografts. Mice were treated three times weekly with intraperitoneal injections of vehicle control and/or DIMATE and/or weekly with CDDP. Tumor growth was monitored using a noninvasive imaging approach involving luciferin injection (4 mg/mouse) and imaging with an IVIS Spectrum instrument. In addition, tumor growth was quantified by CT imaging.

### Bioinformatic and statistical analyses

The following bioinformatic tools were used to analyze the functions of ALDHs in NSCLC biological processes: TCGA, cBioPortal [23], Oncomine [50, 51]; Human Protein Atlas [24]; Kaplan–Meier plotter [52, 53] and GEO [54, 55].

Kaplan–Meier plotter and the R program were used to evaluate the clinical relevance of ALDHs by linking clinical data to gene expression levels. Kaplan–Meier Plotter was used to confirm disease prognosis and OS times. Transcriptome microarray datasets were downloaded from the TCGA and GEO databases under the accession numbers GSE8894 (*N* = 138), GSE19188 (*N* = 91), GSE3141 (*N* = 111), GSE31210 (*N* = 246), GSE4573 (*N* = 130), GSE14814 (*N* = 133), GSE29013 (*N* = 55), GSE37745 (*N* = 196), GSE30219 (*N* = 293), GSE31908 (*N* = 30), GSE43580 (*N* = 150), GSE50081 (*N* = 181), caArray (*N* = 443), and TCGA (*N* = 133). All datasets were transformed to a common scale and precision. Normalized raw transcriptome data were subsequently reanalyzed to evaluate the associations between *ALDH1A1, ALDH1A3,* and *ALDH3A1* expression and clinicopathological variables using the nonparametric Fisher’s exact test. Analysis was performed with the R program [56].

The flow cytometric analysis, cell viability assay, and in vivo tumor growth results were analyzed by a paired *t-*test. *P* < 0.05 was considered statistically significant.

## Supplementary information

Supplementary Information

Supplementary Fig S1

Supplementary Fig S2

Supplementary Fig S3

Supplementary Fig S4

Supplementary Fig S5

Supplementary Fig S6

Supplementary Table S1

Supplementary Table S2

Supplementary Table S3

Supplementary Table S4

Supplementary Table S5
