# Supplementary material for: Dual disruption of aldehyde dehydrogenases 1 and 3 promotes functional changes in the glutathione redox system and enhances chemosensitivity in nonsmall cell lung cancer
Source: Oncogene. 2020 Feb 3;39(13):2756–71. doi: 10.1038/s41388-020-1184-9 (PMC7098886; doi:10.1038/s41388-020-1184-9)
Supplement: Supplementary file 1 — Supplementary Information [file 41388_2020_1184_MOESM1_ESM.docx]

**Supplementary Methods**

***Real-Time PCR analysis***

### Total RNA extraction was performed using Tryzol reagent (Invitrogen) following the manufacturer's instructions. Total RNA was reverse-transcribed into cDNA using M-MLV Reverse Transcriptase (Thermo Fisher) and oligo random hexamers. The cDNA was subjected to quantitative PCR analysis using SYBR Green PCR Master Mix Kit (Applied Biosystems) and the LightCycler 480 system (Applied Biosystems, Monza, Italy) with. The primer sequences to analyze the different ALDHs isoforms were as follow: hALDH1A1 (Fw- TTGGAAATCCTCTGACCCCA, Rv- CCTTCTTTCTTCCCACTCTC), hALDH1A3 (Fw- TTTTCATCGACCTGGAGG, Rv- GACGTTGTCATCTGTGGG), hALDH3A1 (Fw-CACATCACCTTGCACTCTCT, Rv- AGCTCTTCTTGCCATGGT). The most stable housekeeping genes used for normalization were selected with GeNorm software. For these housekeeping genes the primer sequences used were: hVIM (Fw- CCAGCTAACCAACGACAA, Rv- AAGCATCTCCTCCTGCAA), hACTB (Fw- AGCATCCCCCAAAGTTCA, Rv- CCTGTAACAACGCATCTC), hSF3A1 (Fw- TAAGCCAGTTGTGGGGAT, Rv- GGGGTTGTTGATCTCGTT).

***Apoptosis by annexin V/propidium iodide staining***

Cells were analyzed for phosphatidylserine exposure using the annexin-V FITC/propidium iodide (PI) double-staining method (Apoptosis detection kit, BD biosciences, San Jose, CA, USA). Cells were seeded in 6-well plates and treated with or without DIMATE for 6 and 12 hours. After treatment, cells were washed and collected in 100 μL binding buffer with Annexin (1 mg/mL) and propidium iodide (1 mg/mL) following the manufacturer’s instructions. The fluorescence signal was measured using a flow cytometer (FACSCalibur, BD Bioscience, Le-Pont-de-Claix, France) and the data were analyzed using CELLQuest software (BD Biosciences).

For the assessment of DIMATE-induced apoptosis in the CDDP-resistant HCC827 cell line, cells were pre-treated with CDDP (10 µM) for 24 hours to induce CDDP-mediated augmentation of intracellular ROS levels. After treatment, cells were incubated with 2',7' –dichlorofluorescein diacetate (DCFDA, Thermo Fisher) for 20 minutes and the fluorescent signals were detected via flow cytometry. Cells with moderate and high signals of DCFDA were separated using a fluorescence-activated cell sorter (FACSAria, BD Biosciences). Cell subpopulations with low-, moderate- or high ROS levels were seeded separately into 24-well plates for adhesion and treated with or without DIMATE (10 µM) for 12 hours. For assessment of apoptosis, residual DCFDA signal was quenched with trypan blue and cells were stained with Annexin V and PI as described above.

***MDA quantification***

Cells were seeded in 96-well plates and treated with DIMATE (5 µM) for 12, 24, 36 and 48 hours. Quantification of MDA was performed using an OxiSelect™ MDA-Adduct Competitive ELISA (Cell Biolabs, San Diego, CA, USA) following the manufacturer’s protocol.

***ROS quantification***

Intracellular ROS levels were measured using Total ROS/superoxide detection kit (Enzo life science, Villeurbanne, France) following the manufacturer’s instructions. The assay uses specific ROS/RNS probes that upon reaction with ROS and RNS species are oxidized rapidly to highly fluorescent compounds. The experimental tests were performed in cells treated with or without DIMATE (1 µM and 5 µM). ROS inducer PCN (500 µM) and ROS inhibitor NAC (10 mM) were used as the positive and negative experimental control, respectively. Briefly, cells (2x 10^4^) were seeded in 96-well black/clear bottom plates in phenol free-media supplemented with 10% FBS, and incubated with or without DIMATE for 6 hours or with the different control agents for 20 min. After treatment, ROS Detection Solution was added, and cells were incubated for 30 min at 37^0^C in the dark. Fluorescence was detected using an Appliskan fluorescence microplate reader (Thermo Scientific) (Ex/ Em= 488/520 nm and Ex/ Em= 550/610 nm). Data were expressed as the change in arbitrary fluorescence units produced from equal number of cells and normalized to the total protein input.

***Mass spectrometry identification of HNE-protein adducts***

Cells were incubated with or without DIMATE (5 uM) for 24 hours and lysed in RIPA buffer (50 mM Tris–HCl pH 7.9, 1% NP-40, 0.1% SDS, 150 mM NaCl, 1 mM EDTA and protease inhibitor cocktail (Calbiochem)). Cell lysates were precleaned with protein G-conjugated agarose beads (GE Healthcare, Limonest, France) and used for immunoprecipitation with 2 μg of HNE (AbCam, Cambridge, UK), or mouse IgG antibody as the experimental control. The immunoprecipitated samples from each condition were separated in “twin” SDS-PAGE gels. One gel was transferred to nitrocellulose and probed with HNE antibody; the second gel was stained with Sypro Ruby Stain (Bio-Rad). Differential lanes were sliced into fractions and in-gel digested with trypsin (Sequencing grade, Promega). After digestion, samples were desalted using ZipTip C18 with resin (Millipore, Temecula, CA, USA), dried, and resuspended in 0.1% trifluoroacetic acid, 2% acetonitrile. Mass spectrometry and data analyses were performed in the core facility of Vall d’Hebron Institute of Oncology (VHIO, Barcelona) using nanospray ionization (ESI), with tandem mass spectrometry (ESI-MS/MS) as previously described [1]. MS/MS spectra were searched against Proteome Discoverer (version 1.3.0.339, Thermo Fisher Scientific) using MASCOT (version 2.3, Matrix Science) and cross-correlated with SWISS-PROT accession numbers for final protein identification.

***Wes capillary electrophoresis immunoassay***

For protein expression analysis in siRNA and shRNA assays, cells were lysed in RIPA buffer containing phosphatase and protease inhibitors (Calbiochem). Total protein extracts were separated via a Wes capillary electrophoresis immunoassay (Simple Western system, Bio-Techne, CA, USA) [2]. Wes was performed according to the manufacturer’s instructions using a 12-230 kDa separation module and the default instrument settings: stacking and separation at 475 V for 30 min, blocking reagent incubation for 5 min, primary and secondary antibody incubation for 30 min each, and luminol/peroxide chemiluminescence detection for ~15 min. Antibodies used in these studies are listed in **Supplementary Table S4.**

**References**

1. Ferrer-Mayorga G, Alvarez-Diaz S, Valle N, De Las Rivas J, Mendes M, Barderas R *et al.* Cystatin D locates in the nucleus at sites of active transcription and modulates gene and protein expression. J Biol Chem. 2015; 290: 26533-26548.

2. Chen JQ, Heldman MR, Herrmann MA, Kedei N, Woo W, Blumberg PM *et al.* Absolute quantitation of endogenous proteins with precision and accuracy using a capillary Western system. Anal Biochem. 2013; 442: 97-103.

**Supplementary Figure and Table legends**

**Fig. S1.** Transcriptional profile of ALDH1A1, ALDH1A3 and ALDH3A1 in NSCLC cell lines. **a** Real-time PCR analysis of the endogenous expression of ALDH1A1, ALDH1A3 and ALDH3A1 in the normal human bronchial epithelial BEAS-2B cells and the fourteen NSCLC cell line panel. SF3A1, VIM and ACTB were used as internal reference genes for the normalization of the data. **b** Scatterplots of DNA copy numbers versus log2 mRNA gene expression indicating mRNA upregulation across the different cell lines as in **a** (except BEAS-2B cells for which data was not available). NRASmut (green, square), KRASmut (blue, triangle), EGFRmut (red, circle). Data were retrieved from Cancer Cell Line Encyclopedia (<https://portals.broadinstitute.org/ccle>).

**Fig. S2.** Apoptosis and MDA accumulation induced by pharmacological inhibition of class 1 and 3 ALDHs with DIMATE. **a** Dot plots indicating Annexin V-FITC and PI staining in H1650 and H1975 cells treated with DIMATE (5 µM) for 6 and 12 hours and obtained via flow cytometry to assess apoptosis. **b** Temporal analysis of MDA-adducts in H1650 and H1975 cells. Cells were exposed to 5 μM DIMATE and MDA levels were measured at the indicated time points by the reaction of MDA with thiobarbituric acid. Bars represent the mean of 5 replicates ± S.D.

**Fig. S3.** ROS-inducing drugs sensitize H460, Hop62 and H2122 cells to DIMATE-induced cell death. **a** Percentage of apoptotic cells in H460 cells treated with 15 µM DIMATE, 100 µM BSO or 50 µM BCNU alone and in the indicated combinations. Apoptosis was determined using flow cytometry. Error bars, S.E.M. (N=3); ***P< 0.001. **b, c** Dose-response curve for the viability of Hop62 and H2122 cells exposed for 48 hours to DIMATE in the presence or absence of PCN. The IC50 values for the different conditions are provided in the graph. The inner panel shows that 40 μM PCN exhibited no cytotoxicity at the indicated incubation times. The error bars indicate the S.D. (N=4).

**Fig. S4.** Generation of stable HCC827 and H460 cell populations with a compromised redox capacity. **a, b** Protein immunodetection using a Wes assay, showing the amounts of GCLC and CAT in H460 and HCC827 cells infected with dox-inducible shRNA targeting GCLC and CAT, respectively. Non-targeting shRNA vector was used as the negative control. α-Tubulin is shown as the protein loading control. **c** Measurement of intracellular GSH levels in H460 cells with GCLC depletion as described in **a**. The data are expressed as percentages relative to non-targeting control cells (***P<0.001). **d** Viability of stable H460 cells expressing dox-inducible GCLC shRNA treated with or without doxycycline for 96 hours. The data are expressed as percentages relative to wild-type cells. No significant differences in viability were observed among the different cell conditions. **e** Viability of HCC827 cells with CAT depletion as described in b and exposed to 100 µM H2O2. The data are presented as percentages relative to untreated cells (**P<0.05). **f** Viability of stable HCC827 cells expressing dox-inducible CAT shRNA treated as described in **d.** No significant differences in viability were observed among wild-type control cells and dox-induced or uninduced cells.

**Fig. S5.** Downregulation of ALDH1A1, ALDH1A3 and ALDH3A1 expression in H460 and HCC827 cell lines. **a, b** Protein immunodetection using a Wes assay, showing the amounts of ALDH1A1, ALDH1A3 and ALDH3A1 in H460 and HCC827 cells, respectively. Scrambled siRNA was used as the negative control. α-tubulin is shown as the protein loading control. **c, d** Viability of H460 and HCC827 cells as in a and b, respectively. The data are expressed as percentages relative to wild-type cells. **e** List of siRNAs used to downregulate ALDH1A1, ALDH1A3 and ALDH3A1 expression.

**Fig. S6.** Apoptosis in cells subpopulations with low-, moderate- and high ROS levels treated with DIMATE. **a** ROS moderate and ROS high cells sorted from CDDP-treated HCC827 cells and assessed for apoptosis following treatment with DIMATE (10 µM) for 12 hours. Percentage of cells is indicated under every plot. Data are representative of three independent experiments. Untreated cells, which show low ROS levels, were used as the experimental control. **b** Protein immune-detection using a Wes assay, showing the amounts of ALDH1A1, ALDH1A3 and ALDH3A1 in ROS low-, ROS moderate- and ROS high HCC827 cells as detailed in a. α-tubulin is shown as the protein loading control.

**Supplementary Table S1**. List of human cell lines used in this study and their characteristics, including sensitivity to DIMATE, and endogenous levels of ROS, GSH and ALDH activity.

**Supplementary Table S2.** Identified proteins with increased 4-HNE levels at 24 hours of treatment with DIMATE.

**Supplementary Table S3**. Summary of combination index values (CI) for DIMATE/CDDP in NSCLC cells.

**Supplementary Table S4.** List of antibodies used in western blotting and Wes capillary electrophoresis.

**Supplementary Table S5.** List of shRNAs used to knockdown GCLC and CAT.
