# Supplementary figures and images for: Dual disruption of aldehyde dehydrogenases 1 and 3 promotes functional changes in the glutathione redox system and enhances chemosensitivity in nonsmall cell lung cancer
Source: Oncogene. 2020 Feb 3;39(13):2756–71. doi: 10.1038/s41388-020-1184-9 (PMC7098886; doi:10.1038/s41388-020-1184-9)

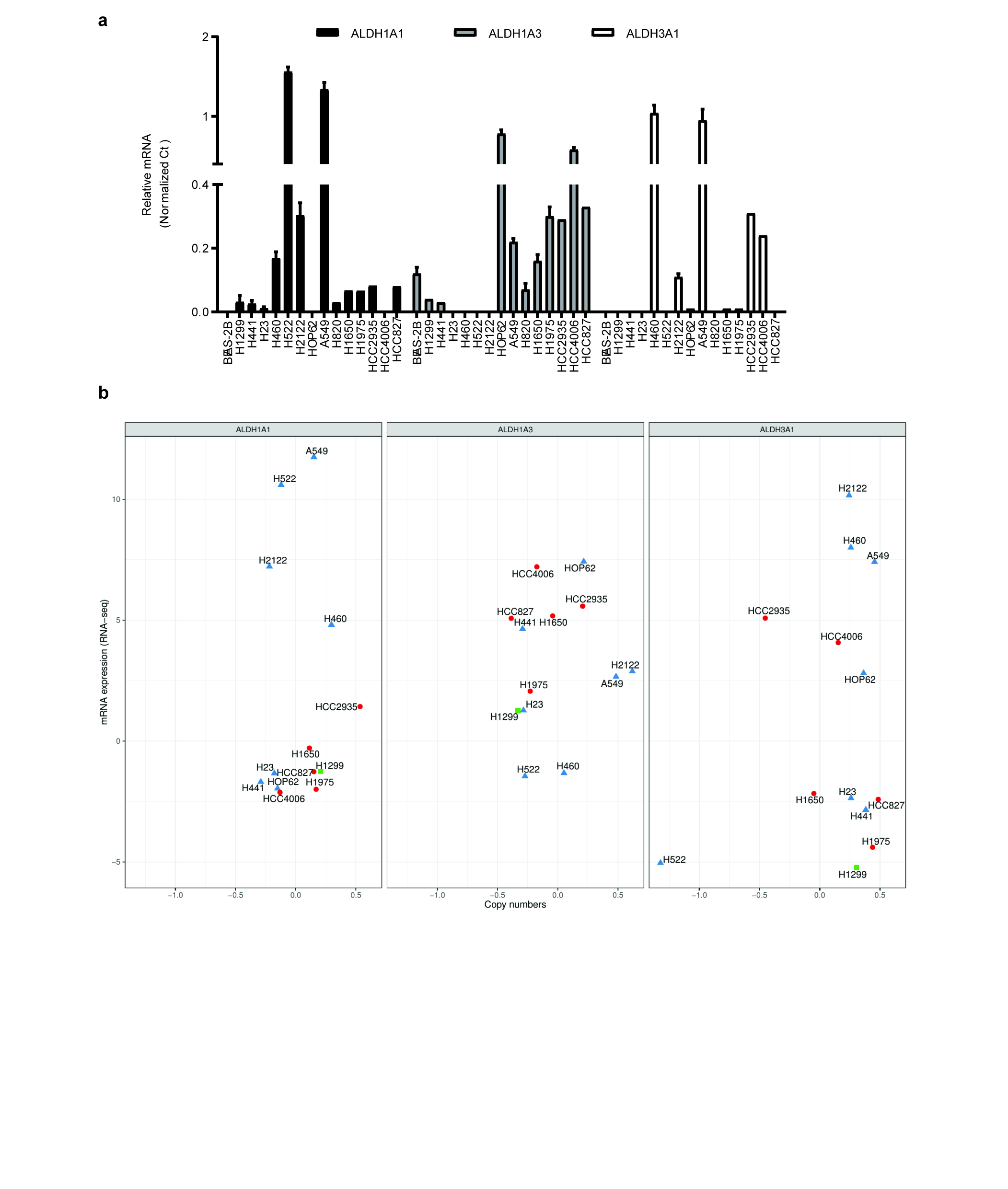

Supplement: Supplementary file 2 — Supplementary Fig S1 [file 41388_2020_1184_MOESM2_ESM.tif]

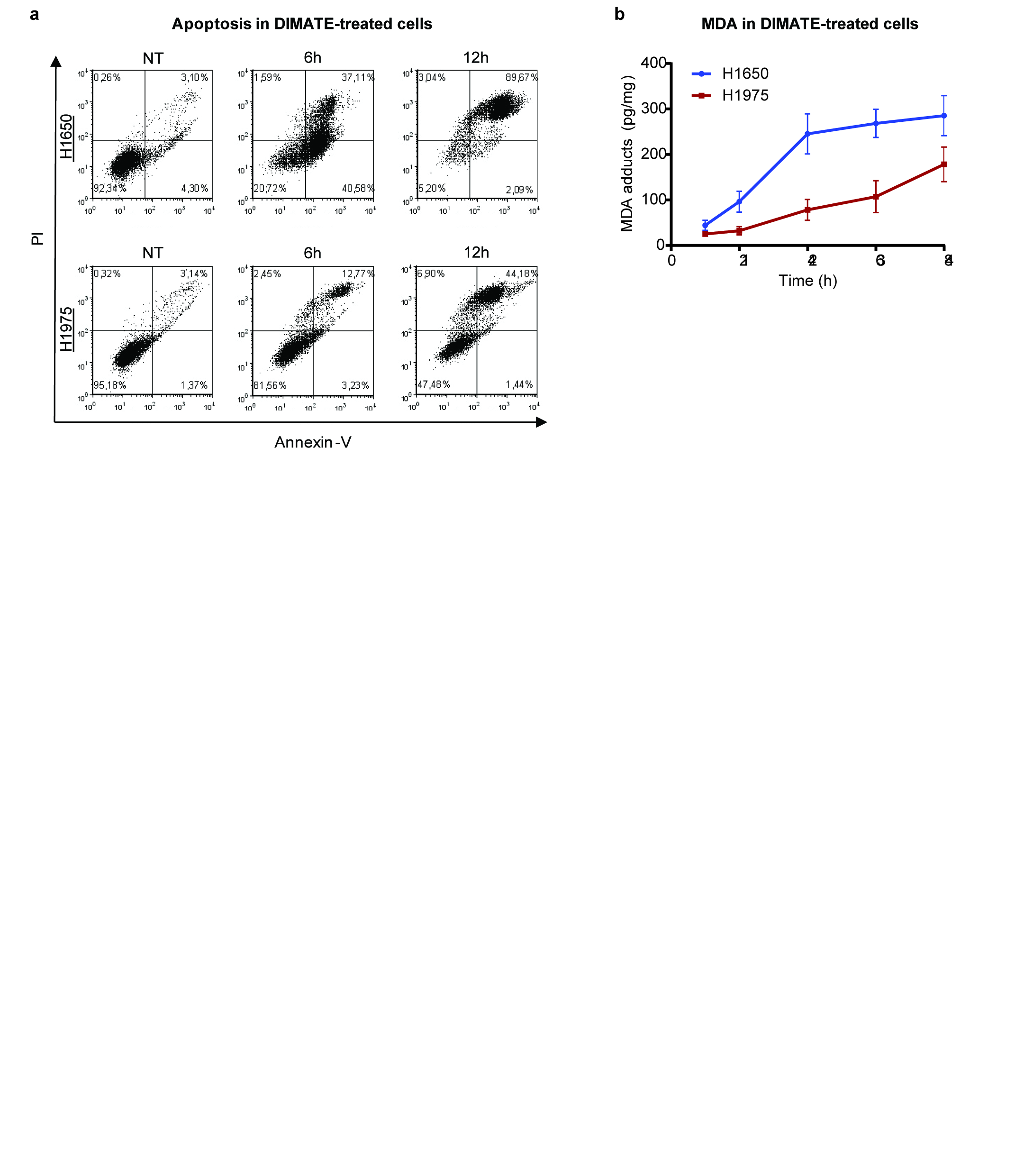

Supplement: Supplementary file 3 — Supplementary Fig S2 [file 41388_2020_1184_MOESM3_ESM.tif]

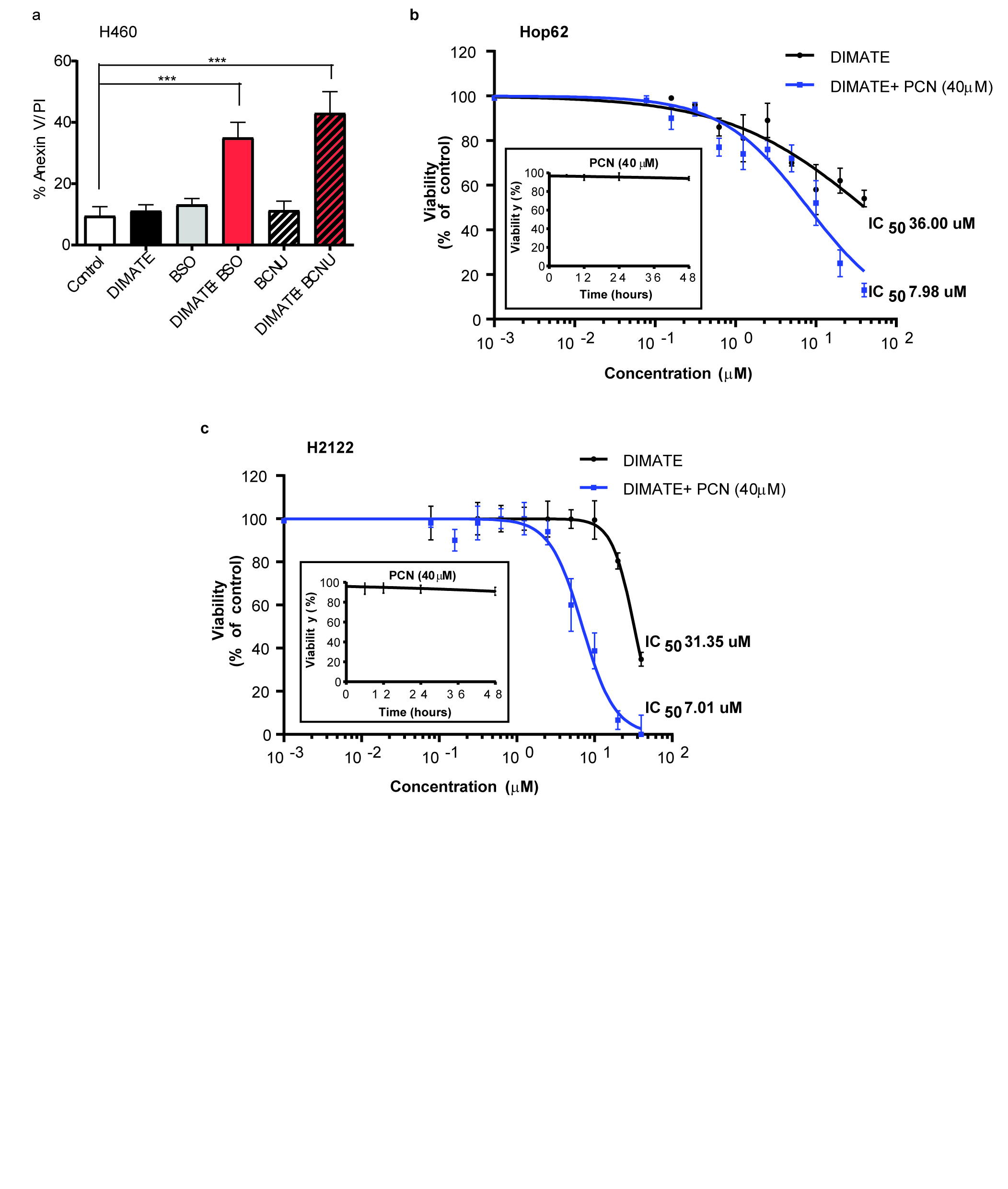

Supplement: Supplementary file 4 — Supplementary Fig S3 [file 41388_2020_1184_MOESM4_ESM.tif]

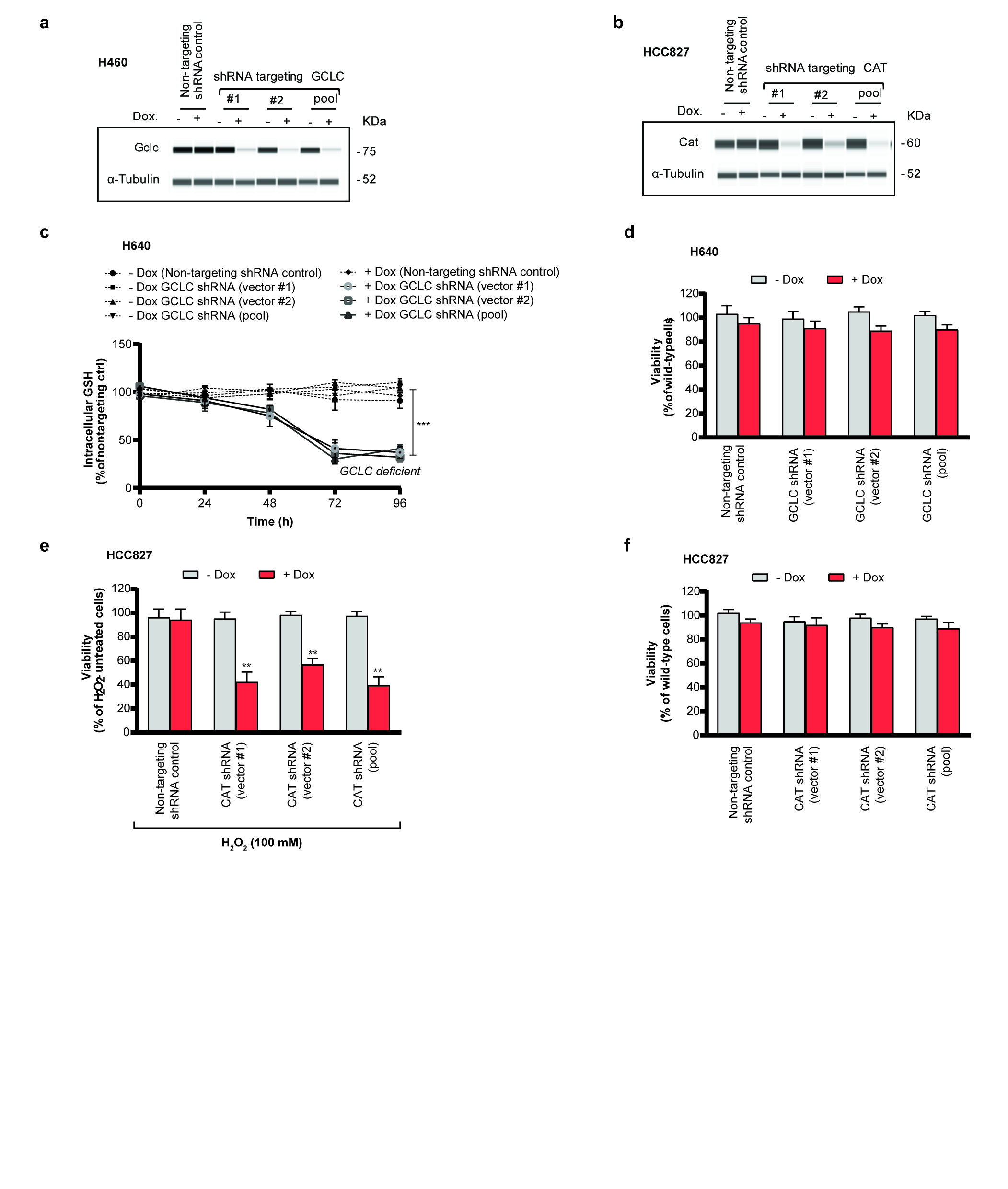

Supplement: Supplementary file 5 — Supplementary Fig S4 [file 41388_2020_1184_MOESM5_ESM.tif]

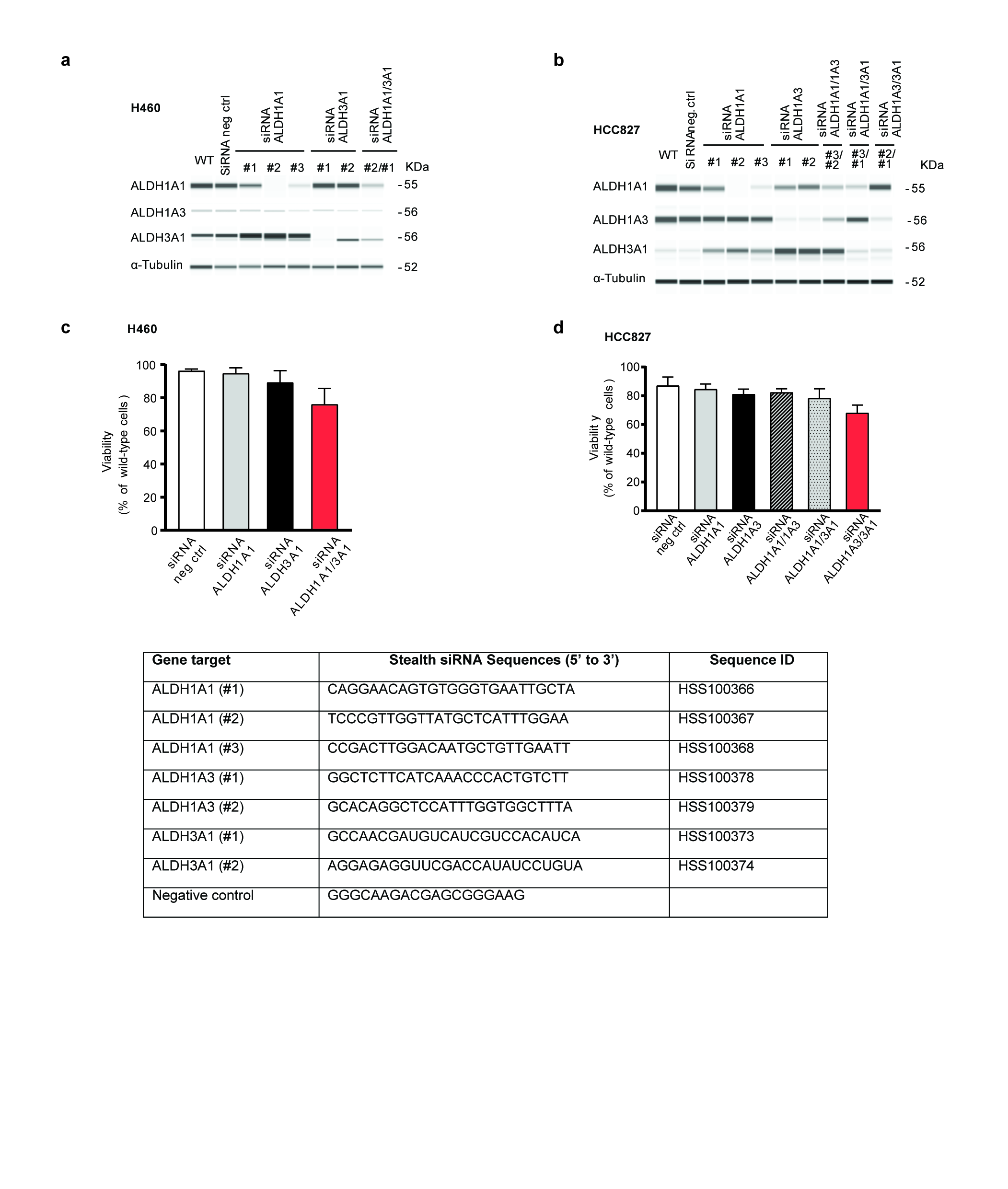

Supplement: Supplementary file 6 — Supplementary Fig S5 [file 41388_2020_1184_MOESM6_ESM.tif]

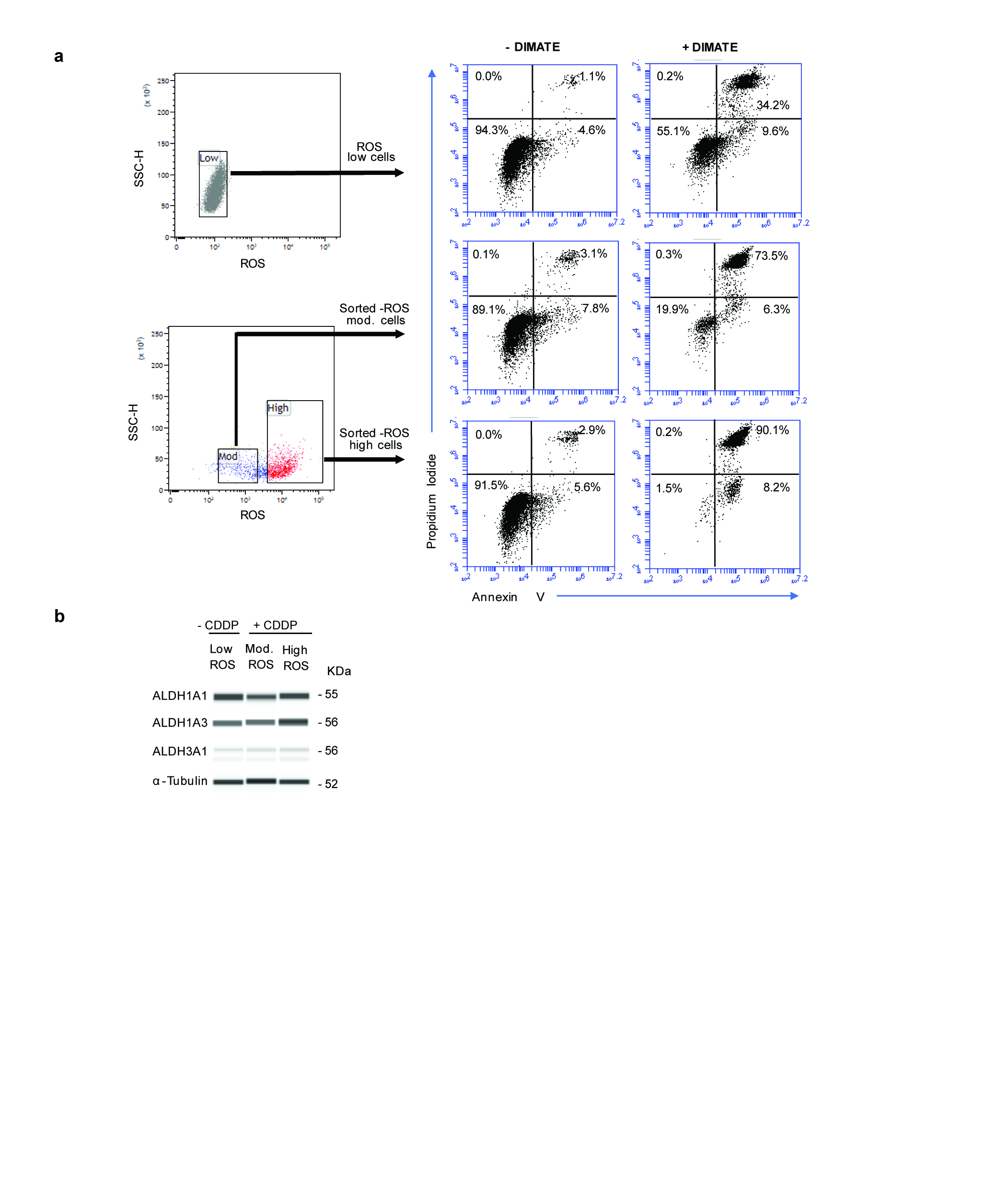

Supplement: Supplementary file 7 — Supplementary Fig S6 [file 41388_2020_1184_MOESM7_ESM.tif]
