# Supplementary material for: Dual disruption of aldehyde dehydrogenases 1 and 3 promotes functional changes in the glutathione redox system and enhances chemosensitivity in nonsmall cell lung cancer
Source: Oncogene. 2020 Feb 3;39(13):2756–71. doi: 10.1038/s41388-020-1184-9 (PMC7098886; doi:10.1038/s41388-020-1184-9)
Supplement: Supplementary file 8 — Supplementary Table S1 [file 41388_2020_1184_MOESM8_ESM.docx]

**Supplementary Table S1.** List of human cell lines used in this study and their characteristics, including sensitivity to DIMATE, and endogenous levels of ROS, GSH and ALDH activity.

| **Cell lines** | **Characteristics** | **DIMATE**  **(IC50, µM)** | **ROS**  **(RFU x103)** | **GSH**  **(µmol/106)** | **ALDH 1**  **activity** | **ALDH 3**  **activity** |
| --- | --- | --- | --- | --- | --- | --- |
| **BEAS-2B** | Immortalized human bronchial epithelial cells. | 50.00 | 10.40± 0.26 | 51.33 | 7.03± 0.16 | - |
| **H1299**** | Human NSCLC; NRAS (p.Q61K), TP53 partial deletion. | 13.03 | 11.87± 0.31 | 46.81 | 14.21± 0.46 | - |
| **H441**** | Human NSCLC, papillary adenocarcinoma; KRAS (p.G12V), TP53 (p.R158L), lack of PTEN. | 11.71 | 28.98± 0.12 | 57.06 | 6.39± 0.98 | - |
| **H23**** | Human NSCLC, adenocarcinoma; KRAS (p.G12C), TP53 (p.M153I (+) M246I), STK11 (p.W332*). | 11.80 | 22.88± 0.53 | 91.01 | 4.67± 0.20 | - |
| **H460** | Human NSCLC, KRAS (p.Q61H), PI3KCA (p.E545K), STK11 (p.Q37X). | 46.50 | 38.65± 0.33 | 136.21 | 5.29± 0.15 | 41.1± 0.35 |
| **H522** | Human NSCLC, adenocarcinoma; TP53 (p. P191fs), SMARCA4 (p. P270fs). | 6.70 | 22.55± 0.48 | 58.55 | 11.48± 0.49 | - |
| **H2122**** | Human NSCLC, KRAS (p.G12C), TP53 (p. Q16L (+) C176F), KEAP-1 (170-204 del). | 31.35 | 10.03± 0.27 | 63.61 | 18.30± 0.39 | 48.6± 0.52 |
| **Hop62**** | Human NSCLC, adenocarcinoma; KRAS (p.G12C). | 36.00 | 11.24± 0.22 | 46.37 | 14.14± 0.64 | 11± 0.18 |
| **A549** | Human NSCLC, alveolar basal epithelial adenocarcinoma; KRAS (p.G12S), STK11 (p.Q23X). | 10.11 | 40.53± 0.37 | 92.47 | 11.05± 0.04 | 43.8± 0.46 |
| **H1650** | Human NSCLC, bronchioloalveolar carcinoma; EGFR (p.E746_A750del), PTEN null. | 1.36 | 33.82± 0.54 | 64.26 | 14.13± 0.50 | 7.3± 0.12 |
| **H1975** | Human NSCLC, adenocarcinoma; EGFR (p.T790M (+) p.L858R), TP53 (p.R273H). | 1.62 | 31.87± 0.46 | 63.59 | 11.24± 0.37 | 7.7± 0.09 |
| **HCC 827** | Human NSCLC, adenocarcinoma; EGFR (p.E76_A750del), TP53 (p.V218delV). | 24.98 | 16.70± 0.28 | 73.22 | 13.42± 0.54 | - |
| **HCC 2935** | Human NSCLC, adenocarcinoma; EGFR (p.E749_T751 del (+) S752I). | 0.97 | 37.24± 0.50 | 46.74 | 12.97± 0.33 | 34± 0.33 |
| **HCC4006** | Human NSCLC, adenocarcinoma; EGFR (p-L747_E749del (+) A750P). | 2.61 | 34.92± 0.47 | 46.84 | 11.37± 0.45 | 29.3± 0.28 |
| **H820**** | Human NSCLC, adenocarcinoma; EGFR (p.T790M), TP53 (p.T284P), TGFBR3 (p.D663H). | 0.58 | 37.01± 0.44 | 34.72 | 11.03± 0.36 | 6.8± 0.10 |
| **LXA-02*** | Human NSCLC; KRAS (p.G12D). | 1.95 | 41.66± 0.37 | 79.98 | 8.34± 0.27 | 14.6± 0.13 |
| **LXA-07*** | Human NSCLC; KRAS (p.G12C). | 2.11 | 39.41± 0.41 | 83.15 | 11.08± 0.27 | - |
| **LXA-08*** | Human NSCLC; KRAS (p.G12A), TP53 (p.R273L). | 6.18 | 32.71± 0.40 | 94.69 | 13.21± 0.34 | - |
| **LXA-9*** | Human NSCLC; EGFR (p.E746_T750del). | 38.06 | 45.80± 0.57 | 129.93 | 18.67± 0.36 | - |
| **LXA-11*** | Human NSCLC; KRAS (p.G12S). | 37.55 | 32.79± 0.33 | 132.06 | 22.77± 0.42 | - |
| **LXA-14*** | Human NSCLC; KRAS (p.G12C); TP53 (p.Y126C). | 2.92 | 28.25± 0.28 | 65.18 | 13.58± 0.22 | - |
| **LXA-15*** | Human NSCLC; KRAS (p.G12D). | 2.00 | 36.60± 0.39 | 90.02 | 6.99± 0.12 | - |
| **LXA-16*** | Human NSCLC; EGFR (p.L878R). | 33.45 | 39.52± 0.41 | 134.43 | 21.12± 0.22 | - |
| **LXA-20*** | Human NSCLC; EGFR (p.E749_T751del). | 1.63 | 43.64± 0.37 | 81.50 | 9.86± 0.34 | - |
| **LXA-22*** | Human NSCLC; EGFR (p.E746_T749del) | 2.41 | 27.81± 028 | 45.34 | 10.69± 0.26 | - |
| **LXA-23*** | Human NSCLC; EGFR (p.E746-T750del) | 1.08 | 44.33± 0.38 | 69.47 | 10.98± 0.36 | - |
| **LXA-25*** | Human NSCLC; EGFR (p.L858R) | 12.69 | 29.19± 0.20 | 96.22 | 15.07± 0.29 | - |
| **MDA-MB-231** | Human breast epithelial adenocarcinoma; BRAF (p.G464V), TP53 (p.R280K). | 2.68 | 20.453± 889 | - | 33.95± 94 | - |

*Patient-derived xenografts; NSCLC- Non small cell lung carcinoma.

**Cell lines kindly provided by Dr. Yokota Jun´s laboratory (IGTP, Barcelona, Spain).
