# Supplementary material for: Dual disruption of aldehyde dehydrogenases 1 and 3 promotes functional changes in the glutathione redox system and enhances chemosensitivity in nonsmall cell lung cancer
Source: Oncogene. 2020 Feb 3;39(13):2756–71. doi: 10.1038/s41388-020-1184-9 (PMC7098886; doi:10.1038/s41388-020-1184-9)
Supplement: Supplementary file 9 — Supplementary Table S2 [file 41388_2020_1184_MOESM9_ESM.docx]

**Supplementary Table S2.** Identified proteins with increased 4-HNE levels at 24 hours of treatment with

DIMATE.

| **Protein identified** | **Accession #** | **Biological**  **Process** | **Number of peptides** | **Score** | **MW (kDa)** | **pI** |
| --- | --- | --- | --- | --- | --- | --- |
| Alpha-enolase | P06733 | Glycolysis | 36 | 1191.3 | 47.1 | 7.7 |
| L-lactate dehydrogenase | P00338 | Glycolysis | 3 | 70.7 | 36.7 | 9.3 |
| Pyruvate kinase isozymes M1/M2 | P14618 | Glycolysis | 7 | 196.8 | 57.9 | 9.0 |
| Glyceraldehyde-3-phosphate dehydrogenase | P04406 | Glycolysis | 16 | 448.9 | 36 | 9.3 |
| Malate dehydrogenase | P40926 | TCA cycle | 15 | 357.4 | 35.5 | 9.8 |
| Fumarate hydratase | P07954 | TCA cycle | 2 | 84.3 | 54.6 | 9.4 |
| Succinyl-CoA ligase | P53597 | TCA cycle | 5 | 79.3 | 36.2 | 9.9 |
| Electron transfer flavoprotein | P13804 | ETC | 3 | 92.2 | 35 | 8.6 |
| Cytochrome b-c1 complex | P31930 | ETC | 2 | 98.6 | 52.6 | 5.9 |
| Acyl-coenzyme A thioesterase | Q9Y305 | Lipid metabolism | 6 | 128 | 49.9 | 9.6 |
| Aldose reductase | P15121 | Lipid metabolism | 7 | 223.6 | 35.8 | 6.6 |
| Trifunctional enzyme | P55084 | Lipid metabolism | 8 | 217.1 | 51.3 | 10.0 |
| Quinone oxidoreductase | Q08257 | Xenobiotics metabolism | 3 | 84 | 35.2 | 9.2 |
| Carbonyl reductase | P16152 | Xenobiotics metabolism | 5 | 106.6 | 30.4 | 9.5 |
| Ribonuclease inhibitor | P13489 | redox homeostasis | 4 | 156.1 | 49.9 | 4.6 |
| Thioredoxin domain-containing protein 5 | Q8NBS9 | redox homeostasis | 3 | 65.1 | 47.6 | 5.6 |
| Glutathione S-transferase omega-1 | P78417 | redox homeostasis | 2 | 36.7 | 27.5 | 6.3 |
| 6-phosphogluconate dehydrogenase | P52209 | PPP | 10 | 178.9 | 53.1 | 7.0 |
| Transaldolase | P37837 | PPP | 5 | 75.3 | 37.5 | 6.4 |
| E3 ubiquitin-protein ligase | P19474 | Cell cycle | 12 | 292.3 | 54.1 | 6.0 |
| Septin-7 | Q16181 | Cell cycle | 10 | 267.5 | 50.6 | 9.4 |
| Elongation factor 1-alpha 2 | Q05639 | Protein synthesis | 15 | 636.9 | 50.4 | 9.7 |
| Elongation factor 1-alpha 1 | P68104 | Protein synthesis | 13 | 596.1 | 50.1 | 9.7 |
| Eukaryotic translation initiation factor 2 | P05198 | Protein synthesis | 7 | 234.8 | 36.1 | 4.9 |
| 26S protease regulatory subunit 6B | P43686 | Proteolysis | 8 | 140.3 | 47.3 | 5.0 |
| 26S protease regulatory subunit 7 | P35998 | Proteolysis | 7 | 223.7 | 48.6 | 5.6 |
| Proteasome subunit alpha type-1 | P25786 | Proteolysis | 5 | 92 | 29.5 | 6.2 |
| 26S protease regulatory subunit 6A | P17980 | Proteolysis | 4 | 58.6 | 49.2 | 5.0 |
| 26S proteasome regulatory subunit 11 | O00231 | Proteolysis | 3 | 80 | 47.4 | 6.1 |
| Proteasome activator complex subunit 2 | Q9UL46 | Proteolysis | 3 | 53.4 | 27.4 | 5.4 |
| ATP synthase subunit gamma | P36542 | ATP synthesis | 2 | 39.5 | 33 | 9.7 |
| Hsp90 co-chaperone Cdc37 | Q16543 | Protein stabilization | 7 | 123.2 | 44.4 | 5.0 |

TCA, tricarboxylic acid cycle ; ETC, Electron transport chain ; PPP, Pentose phosphate pathway.
