# Supplementary material for: Dual disruption of aldehyde dehydrogenases 1 and 3 promotes functional changes in the glutathione redox system and enhances chemosensitivity in nonsmall cell lung cancer
Source: Oncogene. 2020 Feb 3;39(13):2756–71. doi: 10.1038/s41388-020-1184-9 (PMC7098886; doi:10.1038/s41388-020-1184-9)
Supplement: Supplementary file 10 — Supplementary Table S3 [file 41388_2020_1184_MOESM10_ESM.docx]

**ED50**

**ED75**

**ED90**

**ED95**

**Dm**

**m**

**r**

**Dm**

**m**

**r**

**Dm**

**m**

**r**

HCC827

(2:1)

0.373

0.188

0.095

0.060

synergy

24.15

0.68

0.95

15.02

0.77

0.94

5.12

1.36

0.98

Hop62

(4:1)

0.474

0.252

0.137

0.092

synergy

38.21

0.62

0.96

6.02

0.83

0.98

5.59

1.43

0.97

H2122

(2:1)

0.511

0.416

0.344

0.305

synergy

29.96

1.45

0.97

10.82

1.08

0.97

8.36

1.59

0.99

H460

(7:1)

0.699

0.507

0.457

0.353

synergy

44.07

1.74

0.96

5.67

0.62

0.98

14.4

1.61

0.96

**Combination**

**ratio**

*Parameters*

*Diagnosis of*

*combined effect*

DIMATE

CDDP

DIMATE+ CDDP

*CI*

**Cell line**

(DIMATE: CDDP)

Effect level (ED) indicates the effect level % at which the CI was calculated; m is slope, signifies shapes; Dm is IC50 (µM), signifies potency; and r is linear correlation coefficient, signifies conformity.

CI were calculated from the CI equation algorithms using CompuSyn software. CI=1, <1 and >1 indicate additive effect, synergism and antagonism, respectively.

**Supplementary Table S3.** Summary of combination index values (CI) for DIMATE/CDDP in NSCLC cells
