# Supplementary material for: Dual disruption of aldehyde dehydrogenases 1 and 3 promotes functional changes in the glutathione redox system and enhances chemosensitivity in nonsmall cell lung cancer
Source: Oncogene. 2020 Feb 3;39(13):2756–71. doi: 10.1038/s41388-020-1184-9 (PMC7098886; doi:10.1038/s41388-020-1184-9)
Supplement: Supplementary file 11 — Supplementary Table S4 [file 41388_2020_1184_MOESM11_ESM.docx]

**Supplementary Table S4**. List of antibodies used in western blotting and Wes capillary electrophoresis

| **Antibody name** | **Company** |
| --- | --- |
| anti-HNE | Abcam (Cambridge, UK) |
| anti-ALDH1A1 | R&D Systems (Abingdon, UK) |
| anti-ALDH1A3 | Abgent (San Diego, CA, USA) |
| anti-ALDH3A1 | Sigma-Aldrich (Saint-Quentin-Follovier, France) |
| anti-GCLC | Abcam (Cambridge, UK) |
| anti-GAPDH | Trevigen (Gaithersburg, MD, USA) |
| anti-tubulin | Cell Signalling Tech (Leiden, NL) |
| anti-catalase | Cell Signalling Tech (Leiden, NL) |
| HRP-conjugated secondary antibodies | GE Healthcare (Limonest, France) |
