# Supplementary material for: Dual disruption of aldehyde dehydrogenases 1 and 3 promotes functional changes in the glutathione redox system and enhances chemosensitivity in nonsmall cell lung cancer
Source: Oncogene. 2020 Feb 3;39(13):2756–71. doi: 10.1038/s41388-020-1184-9 (PMC7098886; doi:10.1038/s41388-020-1184-9)
Supplement: Supplementary file 12 — Supplementary Table S5 [file 41388_2020_1184_MOESM12_ESM.docx]

**Supplementary Table S5.** List of shRNAs used to knockdown *GCLC* and *CAT*.

| **Gene target (Clone ID)** | **shRNA Vector** | **Gene target sequence** |
| --- | --- | --- |
| CAT (V3IHSHEG_4834471) | piSMART hEF1a/TurboGFP | CGTCATGGCTTAATGTTTA |
| CAT (V3IHSHEG_9808198) | piSMART hEF1a/TurboGFP | GACATCAGCTTTCTGCGCG |
| GCLC (V3IHSHEG_4996468) | piSMART hEF1a/TurboGFP | GTGTGTTTCCTGGACTGAT |
| GCLC (V3IHSHEG_9269572) | piSMART hEF1a/TurboGFP | TGCTTAGACAGTAGGTTGC |
| Inducible non-targeting control | piSMART hEF1a/TurboGFP | ATCTCGCTTGGGCGAGAGTAAG |
